# Modelling the effect of a nutritional shock on tuberculosis in India

**DOI:** 10.1186/s44263-025-00153-x

**Published:** 2025-06-27

**Authors:** Rebecca A. Clark, Roel Bakker, Peter Alexander, Roslyn Henry, Richard G. White, Pranay Sinha, Rein M. G. J. Houben, C. Finn McQuaid

**Affiliations:** 1 Department of Infectious Disease Epidemiology, TB Modelling Group, TB Centre, and Centre for Mathematical Modelling of Infectious Diseases, London School of Hygiene & Tropical Medicine, Keppel Street, London, WC1E 7HT UK; 2 KNCV Tuberculosis Foundation, The Hague, Netherlands; 3 School of Geosciences, University of Edinburgh, Edinburgh, UK; 4 School of Biological Sciences, University of Aberdeen, King’s College, Aberdeen, UK; 5 Department of Medicine, Section of Infectious Diseases, Boston University Chobanian & Avedisian, School of Medicine, Boston, USA

**Keywords:** Tuberculosis, Nutritional shock, Mathematical modelling

## Abstract

**Background:**

Environmental or social changes and shocks that reduce access to adequate nutrition have potential consequences for tuberculosis (TB), as undernutrition is a major driver of TB incidence and poor TB treatment outcomes.

**Methods:**

We developed a transmission model of TB in India with an explicit body mass index (BMI) strata linked to disease progression and treatment outcomes, calibrated to country-specific TB estimates. We projected nutritional shock scenarios affecting supply chains, similar to those experienced at the beginning of the war in Ukraine, using the LandSyMM food system model, compared to a continuation of previous food system trends. Within each scenario, increases in food, fertiliser, and energy prices were linked to changes in the population BMI distribution by food availability and prices. We estimated the impact on TB incidence and mortality in India between 2022 and 2035 of these nutritional shock scenarios compared to maintenance of prior trends.

**Results:**

The worst-case scenario, involving sustained increases in food, fertiliser, and energy prices, predicted that shocks increasing undernutrition could result in a 5.0% (95% uncertainty interval = 4.4, 5.9) and 4.9% (4.2, 5.9) increase in TB incidence and mortality respectively in India in 2035 compared to continuation of previous food system trends. In this scenario, an additional 1.1 million (0.9, 1.3) TB episodes and 177.5 thousand (144.7, 224.3) TB deaths were predicted to occur between 2022 and 2035.

**Conclusions:**

Shocks affecting the population-level BMI distribution could lead to changes in the burden of TB disease. Our findings suggest that the impact of crises on TB disease may be underestimated if the impacts of external shocks on nutrition are not explicitly considered.

**Supplementary Information:**

The online version contains supplementary material available at 10.1186/s44263-025-00153-x.

## Background

Tuberculosis (TB) disease is the largest cause of infectious disease death globally. In 2023, there were 10.8 million TB episodes, and 1.25 million deaths, of which India accounted for 26% of both [1]. Undernutrition is a key driver of TB [1], with more than one in five TB episodes attributable to undernourishment globally, rising to an estimated one-third to one-half in India [2]. Undernutrition increases the severity of TB disease [3], and leads to poorer treatment outcomes including adverse events, relapse, and death [4–7].

Changes to population-level nutritional status are likely to affect TB burden but are seldom considered in TB modelling. Due to its infectious nature, indirect effects from even short-term nutritional shocks resulting in increased TB burden could have long-term consequences through increasing TB infection transmission. Multiple pre-chemotherapy era ecological studies have shown the effect of reduced nutrition on increases in TB incidence [8]. For example, German blockades of road and water routes in the Netherlands in World War II led to a months-long nutritional shock and resulted in sharp increases in TB incidence and mortality [9]. Furthermore, recent analyses suggested that the Great Chinese Famine in 1958–1962 may have resulted in an increased risk of TB across multiple generations in affected communities. [10] Improvements in living standards have been credited with responsibility for the most rapid declines in TB mortality ever witnessed, even prior to the availability of drugs and the Bacillus Calmette–Guérin (BCG) vaccine [11, 12].

Currently, the majority of countries are not on track to meet End TB Strategy goals to end the TB epidemic [13], and the ramifications of nutritional shocks may further reduce their ability to do so. With high TB burden countries facing a multitude of food security issues, from climate change [14] to COVID-19 [15] and even the war in Ukraine [16], the potential implications for TB burden are critical. We explored the issue by combining food system modelling and TB transmission modelling to evaluate the potential effect of nutritional shocks on TB burden in India (a country with both the largest burden of TB, and a high proportion of TB cases attributable to undernutrition), accounting for long-term effects on TB infection transmission.

## Methods

**Fig. 1:**
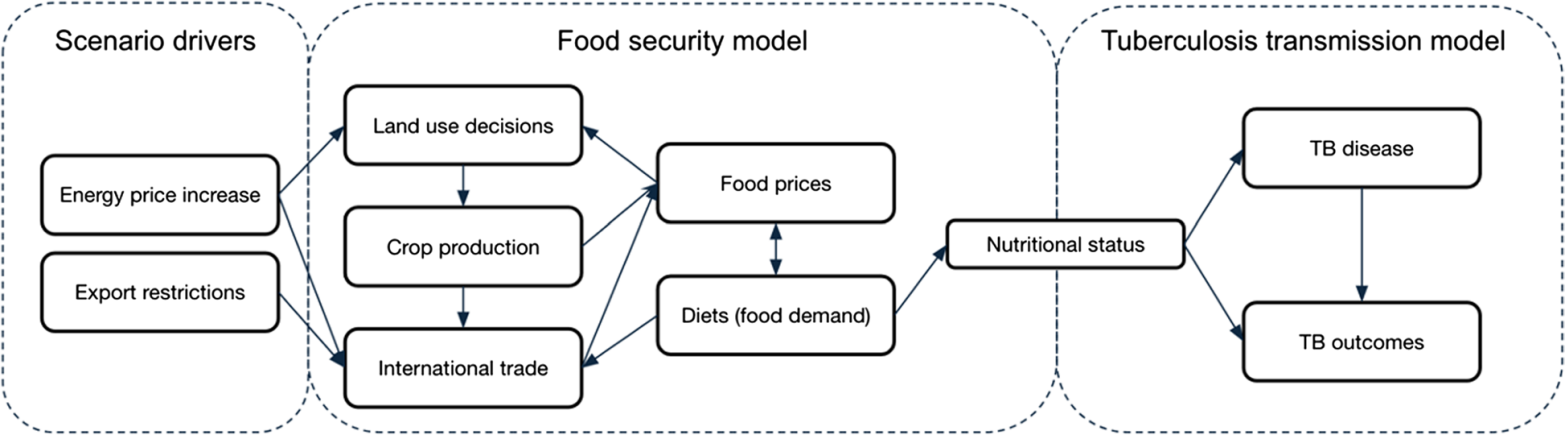
Simplified flowchart showing how increases in energy prices and export restrictions impact nutritional status and TB

Our approach combined a TB infection transmission model [17–19] and a food system model [20] to project future trends in body mass index (BMI), linking these trends to the risk of TB disease and treatment outcomes (Fig. 1). While previously explored in studies of non-communicable diseases [21], this novel approach for TB and infectious disease modelling in general allowed us to consider how important social determinants may change, and the likely effects of those changes on infectious diseases which contain their own feedback mechanisms, providing a foundation for future analyses to build on.

### Data

We obtained demographic data for India from the United Nations Population Division (2019 revision) [22], the Global Health Observatory [23], and India National Family Health Surveys [24–26]. TB disease and infection prevalence estimates were derived from the National TB Prevalence Survey in India 2019–2021 [27]. TB disease incidence, case notifications, mortality, and number of previous treatments were obtained from data available from the World Health Organization (WHO) [28–31]. Estimates of the population attributable fraction (PAF) of undernutrition for TB were obtained from WHO and Bhargava et al. [2, 32, 33] Data to inform the BMI specific-risk of progressing to TB disease and TB treatment outcomes were from Lönnroth et al. [34], Cegielski et al. [35], and Sinha et al. [36], and BMI projections were obtained from the LandSyMM food system model [20]. These projections provided a case study of a nutritional shock, exploring the potential effect of the war in Ukraine on TB in India due to supply chain disruptions and price increases of food, fertiliser and energy.

### Model

We extended a previously published and described age-stratified, compartmental transmission model for TB [17–19]. We defined four BMI strata: moderate to severe thinness (BMI < 17.0 kg/m^2^), mild thinness (17.0 kg/m^2^ ≤ BMI < 18.5 kg/m^2^), normal BMI (18.5 kg/m^2^ ≤ BMI < 25.0 kg/m^2^) and overweight to obese BMI (BMI ≥ 25.0 kg/m^2^). The risk of TB infection [37] and time to diagnosis [38] were assumed to be the same across BMI strata. We varied the risks of progression and reversion to TB disease (similar to methods used by Oxlade et al [39]) and treatment outcomes by BMI strata as in Additional File: Table S3. Further information on our calculations of varying TB risk by BMI strata is included in Additional File 1.

### Calibration

The model was fitted to 15 calibration targets to represent the TB epidemic in India: the TB incidence rate (overall and by age) in 2000 and 2020 [28, 31], the TB mortality rate (overall) in 2000 and 2020 [28], the TB case notification rate (overall and by age) in 2000 and 2020 [29], the TB disease prevalence (overall and for adults) in 2015 and 2021 [27, 40], the TB infection prevalence overall in 2021 [27], and the fraction of asymptomatic TB among active TB [41]. The model was calibrated using history matching with emulation and an Approximate Bayesian Computation using the Markov Chain Monte Carlo method (ABC-MCMC) [42, 43]. We validated our calibration by comparing estimates of PAF due to thinness from our model to recent estimates [2, 32, 33].

### Future scenarios

The LandSyMM food system model [20] was used to simulate four stylised scenarios of BMI trends representing potential nutritional shocks due to the war in Ukraine as in Alexander et al. [16]: (a) a counterfactual *No Shocks* scenario where energy and fertiliser prices and exports conditions were maintained at 2021 levels, (b) an *Export Restriction Shock* scenario that restricted food exports from Russia and Ukraine leading to increases in the price of food grains worldwide, (c) an *Energy Price Shock* scenario that imposed an increase on energy and fertiliser prices, and (d) an *Export and Energy Shocks* scenario that combined both energy price increases and food export restriction shocks.

The mechanism by which food availability and price shocks influence the variations in BMI is as in Alexander et al. [16] and described in detail in Additional File 1: Sect. 2 and 3. In brief, price increases are reflected in shifts in demands of certain types of food, which lead to changes in calorie intake, and consequently, BMI.

We assumed that all shocks were imposed in 2022 and remained in place until the end of the simulations in 2035 and that the quality and coverage of current TB interventions remained constant after 2019, with no new strategies or tools introduced.

### Outcomes

We estimated the TB incidence and mortality rates and the number of TB episodes and deaths for each year from 2022 to 2035 for each of the scenarios. We estimated the relative difference in incidence and mortality rates in 2035 for each scenario compared to the *No Shocks* scenario, and the cumulative difference in the number of TB episodes and deaths between 2035 and 2022 compared to the *No Shocks* scenario.

## Results

The *No Shocks* scenario fit all calibration targets with at least 1000 parameter sets (Additional File 1: Fig. S2). The model estimate of the PAF for undernutrition in 2019 was 18.4% (95% uncertainty interval = 17.8, 19.1), compared to 45.2% (17.0, 71.0) estimated by Bhargava et al. for India specifically.

In the *No Shocks* scenario, 8.5% (8.1, 9.1), 12.5% (11.8, 13.4), 62.6% (62.2, 62.7), and 16.4% (15.2, 17.5) of the population were predicted to have moderate to severe thinness, mild thinness, a normal BMI, and an overweight BMI respectively in 2022, compared to 6.3% (6.0, 6.7), 10.0% (9.5, 10.6), 61.9% (61.7, 62.0), and 21.8% (20.8, 22.7) in 2035 (Additional File 1: Table S7). The *No Shocks* scenario predicted 33.7 million (30.5, 37.1) incident TB episodes and 6.6 million (6.1, 7.2) TB deaths between 2022 and 2035, with an estimated 2.1 million (1.8, 2.3) episodes and 398.3 thousand (365.2, 438.8) deaths in 2035 alone (Table 1).

**Table 1:** Number and rate of incident episodes of TB and TB deaths under different model scenarios and relative difference compared to the *No Shocks* scenario

Scenario	Indicator	Number in 2035 in 1000 s (95% UI)	Incremental number in 2035 (vs *No Shocks*) in 1000 s (95% UI)	Cumulative incremental number between 2022–2035 (vs *No Shocks*) in 1000 s (95% UI)	Rate in 2035 per 100,000 (95% UI)	Relative rate difference in 2035 (vs *No Shocks*) in % (95% UI)	Absolute rate difference in 2035 (vs *No Shocks*) per 100,000 (95% UI)
*No Shocks*	Incident episodes of TB	2071.5 (1847.5, 2300.5)	-	-	133.4 (118.9, 148.1)	-	-
	TB deaths	398.3 (365.2, 438.8)	-	-	25.6 (23.5, 28.3)	-	-
*Export Restriction Shock*	Incident episodes of TB	2076.4 (1852.2, 2302.3)	4.2 (− 3.4, 9.7)	37.2 (1.1, 75.2)	133.7 (119.3, 148.2)	0.2 (− 0.2, 0.5)	0.3 (− 0.2, 0.6)
	TB deaths	399.2 (366.1, 439.9)	0.7 (0.1, 1.3)	5.5 (0.3, 13.4)	25.7 (23.6, 28.3)	0.2 (0, 0.3)	0 (0, 0.1)
*Energy Price Shock*	Incident episodes of TB	2171.9 (1938.9, 2410.9)	100.1 (82.9, 116.9)	1034.6 (851.4, 1220.5)	139.8 (124.8, 155.2)	4.8 (4.1, 5.5)	6.4 (5.3, 7.5)
	TB deaths	416.9 (382.3, 459.8)	18.7 (15.1, 21.6)	169.1 (137.7, 201.0)	26.8 (24.6, 29.6)	4.7 (3.9, 5.3)	1.2 (1.0, 1.4)
*Export and Energy Shocks*	Incident episodes of TB	2175.8 (1941.5, 2416.1)	104.0 (88.1, 125.3)	1083.9 (894.0, 1346.4)	140.1 (125.0, 155.6)	5.0 (4.4, 5.9)	6.7 (5.7, 8.1)
	TB deaths	417.8 (382.9, 460.4)	19.6 (16.4, 23.9)	177.5 (144.7, 224.3)	26.9 (24.7, 29.6)	4.9 (4.2, 5.9)	1.3 (1.1, 1.5)

*Abbreviations*: *TB* tuberculosis, *UI* uncertainty interval

In the *Export Restriction Shock* scenario, the population BMI distribution was similar to the *No Shocks* scenario (Additional File 1: Table S7). TB incidence and mortality were slightly higher, with an additional cumulative 37.2 thousand (1.1, 75.2) TB episodes and 5.5 thousand (0.3, 13.4) deaths between 2022 and 2035 compared to the *No Shocks* scenario (Table 1).

In the *Energy Price Shock* scenario, more individuals experienced thinness, while fewer were overweight (Additional File 1: Table S7). TB incidence and mortality increased more than for the *Export Restriction Shock* scenario, with an additional cumulative 1.0 million (0.9, 1.2) TB episodes and 169.1 thousand (137.7, 201.0) deaths between 2022 and 2035 compared to the *No Shocks* scenario (Table 1).

In the *Export and Energy Shocks* scenario, the largest difference in BMI distribution was observed compared to the *No Shocks* scenario (Additional File 1: Table S7). TB incidence and mortality increased more than for the *Export Restriction Shock* and *Energy Price Shock* scenarios combined, with an additional 1.1 million (0.9, 1.3) TB episodes and 177.5 thousand (144.7, 224.3) deaths between 2022 and 2035 compared to the *No Shocks* scenario (Table 1).

The relative difference in the predicted TB incidence and mortality rates in 2035 between the *No Shocks* and three shock scenarios is shown in Table 1 and Fig. 1. The largest predicted changes were observed in the *Export and Energy Shocks*, where in a “worst-case scenario”, TB incidence and mortality could increase by 5.0% (4.4, 5.9) and 4.9% (4.2, 5.9), respectively, in 2035 compared to the *No Shocks* scenario (Table 1). A larger change in incidence and mortality was predicted from the *Energy Price Shock* scenario (~ 4.7–4.8% increase compared to the *No Shocks* scenario) than the *Export Restriction Shock* scenario (~ 0.2% increase compared to the *No Shocks* scenario).

**Fig. 2:**
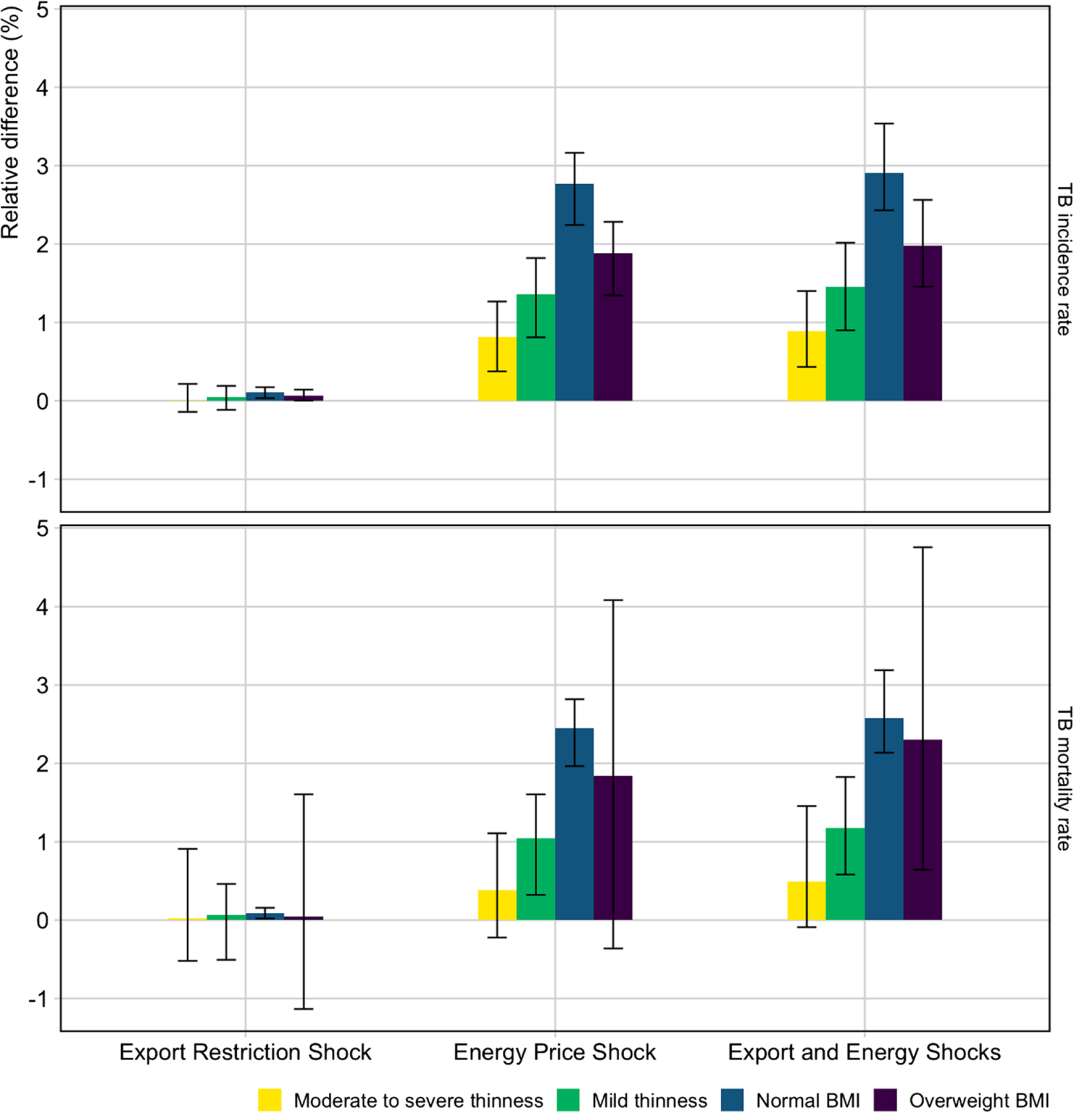
Relative change in TB incidence and mortality rates by BMI in 2035 for each shock scenario

Within each scenario, the relative change in the predicted TB incidence and mortality rates in 2035 varied by BMI strata (Fig. 2). With the *Export and Energy Shocks* scenario, the overall relative increase in the TB incidence rate in 2035 compared to the *No Shocks* scenario was 5.0% (4.4, 5.9) (Table 1). However, this included a 0.9% (0.4, 1.4) increase for moderate to severe thinness, 1.5% (0.9, 2.0) for mild thinness, 2.9% (2.4, 3.5) for normal BMI, and 2.0% (1.5, 2.6) for overweight BMI (Fig. 2). The largest increases in both TB incidence and mortality rates were seen for those with normal BMI.

## Discussion

Nutritional shocks to the global food system, such as energy price increases and export restrictions similar to those due to the war in Ukraine, could have a lasting impact on the burden of TB. In a worst-case scenario, our model predicted that in India alone such shocks could result in a 5.0% (4.4, 5.9) and 4.9% (4.2, 5.9) increase in TB incidence and mortality respectively in 2035. Cumulatively, an additional 1.1 million (0.9, 1.3) people were predicted to develop TB disease, and an additional 177.5 thousand (144.7, 224.3) deaths were predicted to occur between 2022 and 2035.

We evaluated the impact on TB cases and deaths of three scenarios assuming an increase in energy prices and export restrictions, both individually and combined, compared to a *No Shocks* scenario. With decreases in overall BMI from the shock scenarios, we observed increases in the number of people with mild and moderate thinness. Consequently, more TB episodes and deaths were predicted, and the TB incidence and mortality rates were increased in lower BMI strata in the shock scenarios compared to the *No Shocks* scenario.

Our approach combined two modelling frameworks, a TB infection transmission model and the LandSyMM food system model, to both project future trends in BMI and separately to link trends in BMI to TB disease [17–20]. This novel approach allows us to consider both how important social determinants may change, and the likely effects of those changes on infectious diseases which contain their own feedback mechanisms, providing a foundation for future analyses to build on. However, this additional model complexity increases the number of assumptions, running the risk that the model overall becomes less easily interpretable. We also assumed current trends would continue, whereas in reality, scale-up of case-finding and other preventive treatment, intervention coverage is likely to improve with a consequent overall decrease in TB burden.

Our work has limitations. We accounted for differential risk due to BMI, but other factors have been identified that modify the risk of TB. Micronutrients, such as vitamins A and E, have been associated with an increased risk of disease progression independent of BMI [44, 45]. The model may have underestimated TB incidence and mortality as it did not account for TB progression related to reductions in micronutrient intake. Notably, rising food prices may result in reduced food diversity with decreased consumption of vegetables, dairy, and meat and increase the prevalence of micronutrient deficiencies without large BMI reductions. While BMI may be a crude measure of the nutritional deficiencies that lead to an increase in the risk of TB disease, it is the only measure for which there is a strong empirical evidence base [34, 46]. Once more evidence to parameterise the increased risk of TB due to micronutrients is available, future work could extend TB models to include the effect of changes in micronutrients.

The risk of disease progression associated with very low BMI in our model is not well-informed by data, as previous reviews contained no studies with a BMI of less than 17 kg/m^2^ [34]. It is possible that the log-linear relationship between BMI and TB incidence does not hold here. If the TB incidence and mortality risk rises in a non-linear fashion at low BMIs, our model may underestimate the burden of TB in individuals with moderate (BMI 16–17 kg/m^2^) or severe undernutrition (BMI < 16 kg/m^2^) [35]. However, an updated, unpublished review which includes more studies, more settings and participants across a wider range of BMI values (15–35 kg/m^2^) similarly found that the log-linear relationship with BMI holds, suggesting that this may not be the case [47]. More data on the nutritional status of populations and its association with TB, in particular from high TB burden countries, are critical to inform and validate future models of undernutrition and TB.

The primary purpose of this paper was to demonstrate that an understanding of the impact of external shocks on population-level BMI distribution can inform our understanding of the direct impact on TB and that without policy changes to address either the underlying cause of the external shock or the effect, there could be an increase in TB burden. Other work has separately investigated the impact of improving population-level nutritional status or introducing nutritional support for TB-affected households [39, 48, 49]. Future work to further investigate possible mitigation pathways to the effects of external shocks, including societal or political responses that would alleviate or reduce the impact of the food export restrictions or energy price increases, would be useful to inform policy. Additionally, we did not account for the differential impact of disruptions to BMI on the population by aspects such as socioeconomic status, and therefore the resulting population-level effects may be underestimated. For example, the effect of external shocks may be more severe for those with the lowest BMI or low socioeconomic status, which we do not capture in this analysis. More data to parameterise the association between TB, BMI, and socioeconomic status is urgently needed to incorporate this in future models. We did not model the potential impact of changes in BMI on the risk of diabetes mellitus, which may additionally influence the risk of TB [50, 51]. The risk of TB by BMI has also been found to vary by age, but available data to quantify this relationship is sparse, and therefore was not included in our model. However, we did incorporate age-specific BMI distributions and differential risks of TB by age independent of BMI.

## Conclusions

Our analysis demonstrated a stylised exploration of the impact that changes in the population-level distribution of BMI could have on the burden of TB in the country with the largest global burden. Apparently, unconnected events in one region, such as the war in Ukraine, have the potential to worsen the burden of infectious disease in another, such as TB in India, by affecting global food prices. Although we began our study with the assumption that the war in Ukraine would affect food access and availability in India, surplus grain reserves, reductions in grain export in response to rising prices, and a lack of oil boycott in India [52] suggest that despite reduced access to fertilisers [53], observed disruptions may have been more limited than in our model, particularly in the future, and as a consequence the effect on BMI less extreme. However, the effects of issues such as climate change, environmental degradation and food price inflation are more likely to increase undernutrition in India than the scenarios here, and it is vital that we understand this potential effect [54].

Although not intended to inform public health policy directly, our results show the importance of worldwide interactions and cascading risks, where a chain of consequences across systems is distributed globally, worsened through the multiplicative effect of transmission. These and other threats to food security have potentially severe consequences for the ability to end TB in India and globally. If increased vulnerability to TB due to a rise in undernutrition is not explicitly considered, estimates of the impact of crises on TB are at risk of severely underestimating increases in burden.

## Supplementary Information

Additional file 1. Additional File for Modelling the effect of nutritional shocks on tuberculosis in India. This additional file contains detailed methods for the TB transmission model, the climate and food systems models, and the future scenarios used within the manuscript. The additional file also contains epidemiological trends in the *No Shocks* scenario, and further results from the future scenarios.

## Data Availability

The analytic code is publicly available from 10.5281/zenodo.14160777 [55]. The model code used for the future scenarios is publicly available from https://git.ecdf.ed.ac.uk/lul/plumv2/-/tags/RussiaUkrainePaper [56].
