# Supplementary material for: Modelling the effect of a nutritional shock on tuberculosis in India
Source: BMC Glob Public Health. 2025 Jun 27;3:56. doi: 10.1186/s44263-025-00153-x (PMC12203713; doi:10.1186/s44263-025-00153-x)
Supplement: Supplementary file 1 — Additional file 1. Additional File for Modelling the effect of nutritional shocks on tuberculosis in India. This additional file contains detailed methods for the TB transmission model, the climate and food systems models, and the future scenarios used within the manuscript. The additional file also contains epidemiological trends in the No Shocks scenario, and further results from the future scenarios. [file 44263_2025_153_MOESM1_ESM.docx]

**Additional File for *Modelling the effect of nutritional shocks on tuberculosis in India***

Rebecca A. Clark, Roel Bakker, Peter Alexander, Roslyn Henry, Richard G. White, Pranay Sinha,
Rein M.G.J. Houben, C. Finn McQuaid

**Table of Contents**

[ADDITIONAL METHODS 2](#_Toc193965188)

[1. TB transmission model 2](#_Toc193965189)

[1.1 TB model structure and natural history 2](#_Toc193965190)

[1.2 Force of infection equation 4](#_Toc193965191)

[1.3 TB treatment initiation and outcomes 8](#_Toc193965192)

[1.4 Incorporating BMI into the TB model 10](#_Toc193965193)

[1.5 TB model simulation, calibration, and validation 13](#_Toc193965194)

[2. Climate and food systems model 16](#_Toc193965195)

[2.1 LandSyMM 16](#_Toc193965196)

[2.2 Population weight distributions 17](#_Toc193965197)

[3. Future scenarios 18](#_Toc193965198)

[ADDITIONAL RESULTS 19](#_Toc193965199)

[4. Epidemiological trends in the *No Shocks* scenario 19](#_Toc193965200)

[5. Future scenario results 22](#_Toc193965201)

[REFERENCES 29](#_Toc193965202)

#

# ADDITIONAL METHODS

## TB transmission model

We created an age-stratified compartmental differential equation model of tuberculosis (TB) in India, including dimensions for age, TB natural history, and BMI. The age structure is identical to that included in Clark et al.[^1^](https://www.zotero.org/google-docs/?weEEd1) Minor modifications from the Clark et al. natural history structure are described below in section 1.1, the force of infection equation is described in section 1.2, TB treatment is described in section 1.3, and extensions to include four BMI strata, as well as differences in TB progression and treatment, are in section 1.4.

### 1.1 TB model structure and natural history

The TB natural history structure with eight compartments is shown in Figure S1 and has been described previously.[^1^](https://www.zotero.org/google-docs/?Cg5nuo) The infection structure in this model demonstrated a progressive loss of ability to reactivate, with the reactivation rate in the Latent-Fast compartment greater than in Latent-Slow and greater still than in Latent-Zero, where we assumed the rate of reactivation is 0. We did not explicitly consider a self-clearance compartment. We assumed that those in Latent-Fast can fast progress to subclinical disease, or continue to remain latent and transition to Latent-Slow. There was no direct transition between Latent-Fast and Latent-Zero.

Parameters used in the natural history model structure are provided in Table S1, along with their definitions, sources, and information on whether the parameter was fixed or varied during calibration. The parameter ranges provided for the TB natural history parameters were priors fitted during calibration in a Bayesian analysis. We assumed that all values within the prior range were equally likely. The prior ranges were pre-specified based on literature review and were reviewed as new data became available.

We assumed that aspects of TB natural history and mortality varied by age. This was implemented by stratifying certain natural history parameters by age and applying age-specific prior ranges and relative constraints during calibration.[^2^](https://www.zotero.org/google-docs/?fTOa33) Table S2 describes the method used to operationalise the age-varying differences in TB natural history parameters between adults, defined as all ages greater than and equal to 15 years, and children, defined as all ages less than 15 years. For the rate per year of reactivation, relapse, and fast progression to tuberculosis disease, we assumed that the rate for children was less than that for adults. For mortality rates, we assumed the opposite: the rate for children was higher than that for adults. Steps for calculating TB treatment initiation, treatment completion, non-completion, and mortality rates, as well as accounting for public and private sector treatment are described in the Additional File for Clark et. al.[^1^](https://www.zotero.org/google-docs/?dSBcAa)


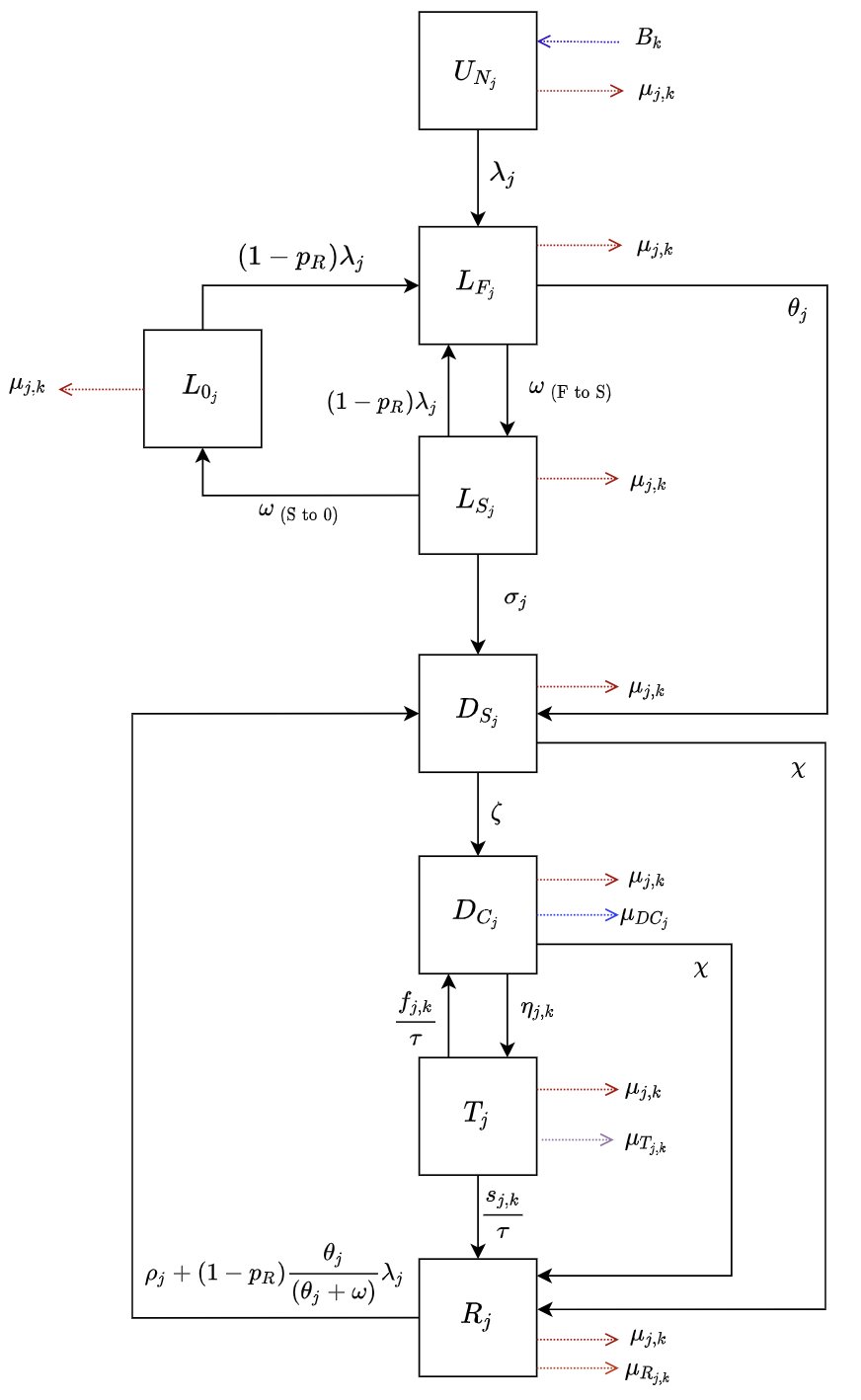


**Figure S1** TB natural history model structure

*Subscript j represents parameters that vary by age, and subscript k represents parameters that vary over time.*

*Abbreviations: U_N_ = Uninfected-Naive; L_F_ = Latent-Fast; L_S_ = Latent-Slow; L_0_ = Latent-Zero, D_S_ = Subclinical Disease; D_C_ = Clinical Disease; T = On-Treatment; R = Recovered.*

### 1.2 Force of infection equation

The equation for the age-specific force of infection ([
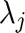
](https://www.codecogs.com/eqnedit.php?latex=%5Clambda_j#0)), or the rate at which Uninfected-Naïve individuals acquire *Mtb* infection in the population, is given below, as in Clark et al. with some minor modifications.^1^ We discounted the force of infection by the proportion of incident cases that are extrapulmonary to account for the fact that they are not infectious, and we also discounted the force of infection to account for the relative reduced infectiousness of subclinical disease compared to clinical disease.

[
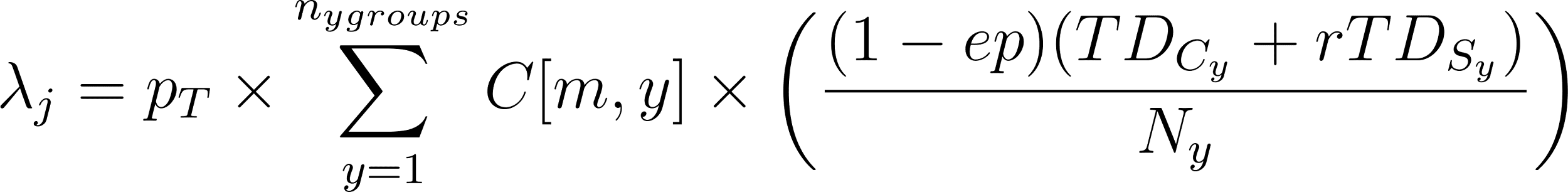
](https://www.codecogs.com/eqnedit.php?latex=%20%5Clambda_j%20%3D%20p_T%20%5Ctimes%20%5Csum_%7By%3D1%7D%5E%7Bn_%7Bygroups%7D%7D%20C%5Bm%2Cy%5D%20%5Ctimes%20%5Cleft%20(%5Cfrac%7B(1-ep)(TD_%7BC_y%7D%20%2B%20rTD_%7BS_y%7D)%7D%7BN_y%7D%20%5Cright)%20#0)

**Table S1** Parameter definitions for the force of infection equation

| **Parameter** | **Definition** |
| --- | --- |
| [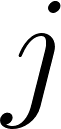](https://www.codecogs.com/eqnedit.php?latex=%20j%20#0) | Age of individual (in years) |
| [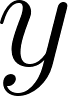](https://www.codecogs.com/eqnedit.php?latex=%20y%20#0) | Age group of contact |
| [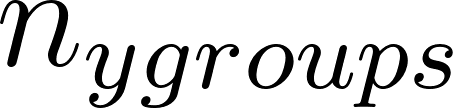](https://www.codecogs.com/eqnedit.php?latex=n_%7Bygroups%7D#0) | Number of contact age groups |
| [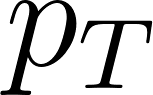](https://www.codecogs.com/eqnedit.php?latex=%20p_T%20#0) | Accounting for the probability of transmission per infectious contact |
| [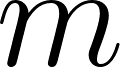](https://www.codecogs.com/eqnedit.php?latex=%20m%20#0) | Age group of individual |
| [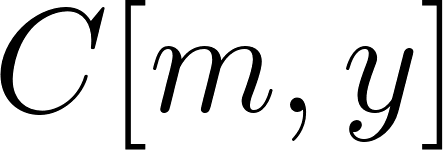](https://www.codecogs.com/eqnedit.php?latex=%20C%5Bm%2C%20y%5D%20#0) | Contact rate between individual of age group [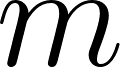](https://www.codecogs.com/eqnedit.php?latex=m#0) and contact of age group [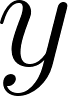](https://www.codecogs.com/eqnedit.php?latex=y#0) from Prem et al.^3^ |
| [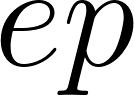](https://www.codecogs.com/eqnedit.php?latex=%20ep%20#0) | Average proportion of tuberculosis cases that are extrapulmonary |
| [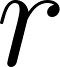](https://www.codecogs.com/eqnedit.php?latex=%20r%20#0) | Proportional reduction in infectiousness from subclinical disease |
| [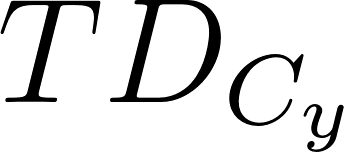](https://www.codecogs.com/eqnedit.php?latex=%20TD_%7BC_y%7D%20#0) | Total population in a clinical disease class in age group [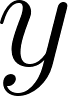](https://www.codecogs.com/eqnedit.php?latex=%20y%20#0) |
| [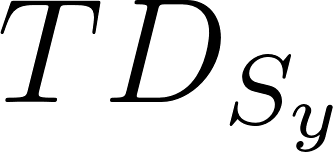](https://www.codecogs.com/eqnedit.php?latex=%20TD_%7BS_y%7D#0) | Total population in a subclinical disease class in age group [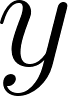](https://www.codecogs.com/eqnedit.php?latex=y#0) |
| [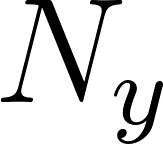](https://www.codecogs.com/eqnedit.php?latex=N_y#0) | Total population alive in age group [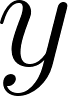](https://www.codecogs.com/eqnedit.php?latex=%20y%20#0) |
| [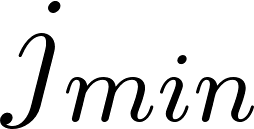](https://www.codecogs.com/eqnedit.php?latex=%20j_%7Bmin%7D%20#0) | Minimum age [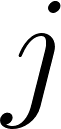](https://www.codecogs.com/eqnedit.php?latex=%20j%20#0) within age group [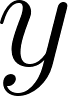](https://www.codecogs.com/eqnedit.php?latex=%20y%20#0) |
| [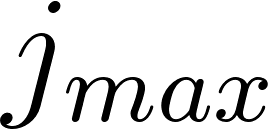](https://www.codecogs.com/eqnedit.php?latex=%20j_%7Bmax%7D%20#0) | Maximum age [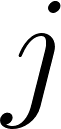](https://www.codecogs.com/eqnedit.php?latex=%20j%20#0) within age group [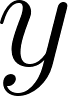](https://www.codecogs.com/eqnedit.php?latex=%20y%20#0) |

Protection against reinfection for those in the Ls, Lf, L0, and R states is represented by pR, which is a proportion between 0.6 and 0.85,^4–8^ and acts to reduce the rate of infection for those with current infection (representing 60–85% protection against reinfection compared to those who are uninfected). Therefore (1-pR) [
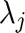
](https://www.codecogs.com/eqnedit.php?latex=%5Clambda_j#0) is the effective reinfection rate for individuals in those states.

**Table S2** India national model parameter values and sources

| **Description** | **Units** | **Symbol** | **Prior** | **Fixed or Varying During Calibration** | **Age Varying** | **Time Varying** | **Source** |
| --- | --- | --- | --- | --- | --- | --- | --- |
| ***Births and deaths (excluding on-treatment mortality)*** | | | | | | | |
| Birth rate | Per year | [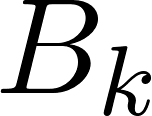](https://www.codecogs.com/eqnedit.php?latex=B_k#0) | United Nations World Population Prospects population estimates and projections | Fixed | No | Yes | ^9^ |
| Background mortality rate | Per year |  | Calculated in the model from United Nations population estimates and projections | Fixed | Yes, age specific mortality rates from demographic dataset | Yes | [^9^](https://www.zotero.org/google-docs/?TGVq1y) |
| Mortality rate for clinical tuberculosis disease | Per person  per year | [***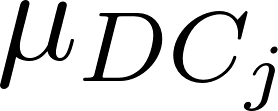***](https://www.codecogs.com/eqnedit.php?latex=%5Cmu_%7BDC_j%7D#0) | (0–0.178) | Varying | Yes, value for children is greater than value for adults | No | [^10^](https://www.zotero.org/google-docs/?tHXTni) |
| Mortality rate post-tuberculosis disease | Per person  per year | [*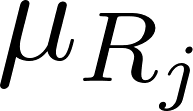*](https://www.codecogs.com/eqnedit.php?latex=%5Cmu_%7BR_j%7D#0) | [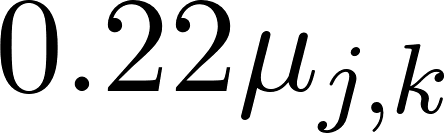](https://www.codecogs.com/eqnedit.php?latex=0.22%5Cmu_%7Bj%2Ck%7D#0) | Fixed relationship | Yes because [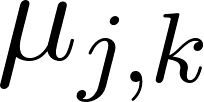](https://www.codecogs.com/eqnedit.php?latex=%5Cmu_%7Bj%2Ck%7D#0) varies | Yes because [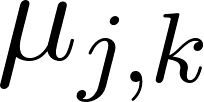](https://www.codecogs.com/eqnedit.php?latex=%5Cmu_%7Bj%2Ck%7D#0) varies | ^11^ |
| ***Natural History*** | | | | | | | |
| Force of infection | Per year | [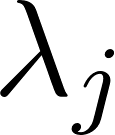](https://www.codecogs.com/eqnedit.php?latex=%5Clambda_j#0) | Fitted | Fixed Equation | Yes, age specific contact rates ^3^ | No | *Calculated* |
| Probability of transmission per infectious contact | - | [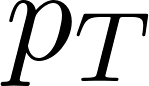](https://www.codecogs.com/eqnedit.php?latex=p_T#0) | (0–0.0068) | Varying | No | No | *Assumed* |
| Fraction of total tuberculosis that is extrapulmonary | - | [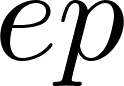](https://www.codecogs.com/eqnedit.php?latex=ep#0) | 0.222 | Fixed | No | No | [^12,13^](https://www.zotero.org/google-docs/?eTc0Pq) |
| Infectiousness of subclinical relative to clinical tuberculosis | - | [*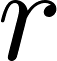*](https://www.codecogs.com/eqnedit.php?latex=r#0) | 0.83 | Fixed | No | No | [^14^](https://www.zotero.org/google-docs/?tHXTni) |
| Rate of fast progression to disease, by age | Per person per year | [*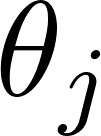*](https://www.codecogs.com/eqnedit.php?latex=%5Ctheta_j#0) | (0.0696–0.111) | Varying | Yes; retained if value for children  was **less** than value for adults. | No | [^15^](https://www.zotero.org/google-docs/?TGVq1y) |
| Rate from L_F_ to L_S_ | Per person per year | [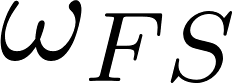](https://www.codecogs.com/eqnedit.php?latex=%5Comega_%7BFS%7D#0) | 0.5 | Fixed | No | No | *Defined* |
| Rate of reactivation from L_S_, by age | Per person per year | [*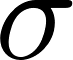*](https://www.codecogs.com/eqnedit.php?latex=%5Csigma#0) | (0.000135–0.00113) | Varying | Yes; retained if value for children was **less** than value for adults. | No | [^15^](https://www.zotero.org/google-docs/?ZIv45I) |
| Rate from L_S_ to L_0_ | Per person per year | [*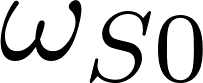*](https://www.codecogs.com/eqnedit.php?latex=%5Comega_%7BS0%7D#0) | (0.0254–0.0467) | Fixed | No | No | [^15^](https://www.zotero.org/google-docs/?izbNsb) |
| Rate of progression from D_S_ to D_C_ | Per person per year | [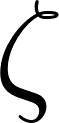](https://www.codecogs.com/eqnedit.php?latex=%20%5Czeta%20#0) | (0–12) | Varying | No | No | *Assumed* |
| Rate of natural cure from D_C_ and D_S_ | Per person per year | [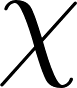](https://www.codecogs.com/eqnedit.php?latex=%5Cchi#0) | (0.10–0.25) | Varying | No | No | [^4,5^](https://www.zotero.org/google-docs/?CyKFIQ) |
| Rate of relapse from R, by age | Per person  per year | [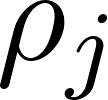](https://www.codecogs.com/eqnedit.php?latex=%5Crho_j#0) | (0.0001–0.07) | Varying | Yes; retained if value for children was **less** than value for adults. | No | [^16–18^](https://www.zotero.org/google-docs/?ep8weA) |
| ***Protection Parameters*** | | | | | | | |
| Protection from reinfection  L_S_, L_F_, L_0_, R | - | [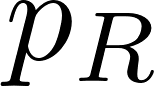](https://www.codecogs.com/eqnedit.php?latex=%20p_R%20#0) | (0.60–0.85) | Varying | No | No | [^4–8^](https://www.zotero.org/google-docs/?pQwJBk) |

###

**Table S3** Operationalising age-varying parameters

| **Parameter** | **Range** | **Age Varying Description** | **Age Scaling Parameter** | **Adults**  **(**[**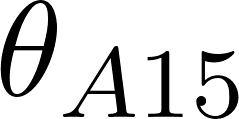**](https://www.codecogs.com/eqnedit.php?latex=%5Ctheta_%7BA15%7D#0)**)** | **Children**  **(**[**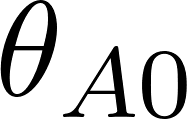**](https://www.codecogs.com/eqnedit.php?latex=%5Ctheta_%7BA0%7D#0)**)** |
| --- | --- | --- | --- | --- | --- |
| [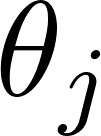](https://www.codecogs.com/eqnedit.php?latex=%5Ctheta_j#0)  Rate per year of fast progression | [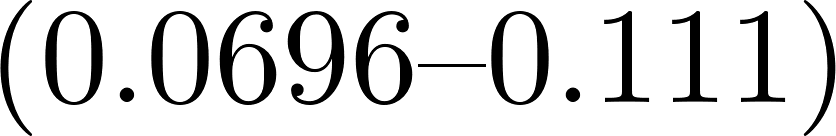](https://www.codecogs.com/eqnedit.php?latex=(0.0696%5Ctextendash0.111)#0) | Retained if value for children was **less** than value for adults | Sample 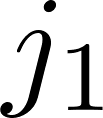  from [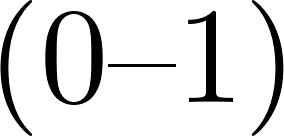](https://www.codecogs.com/eqnedit.php?latex=(0%5Ctextendash1)#0) | Sample [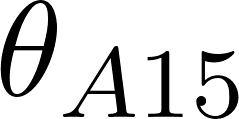](https://www.codecogs.com/eqnedit.php?latex=%5Ctheta_%7BA15%7D#0) from  [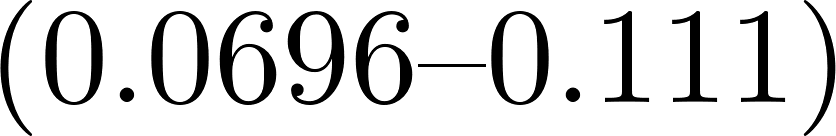](https://www.codecogs.com/eqnedit.php?latex=%20(0.0696%5Ctextendash0.111)#0) | [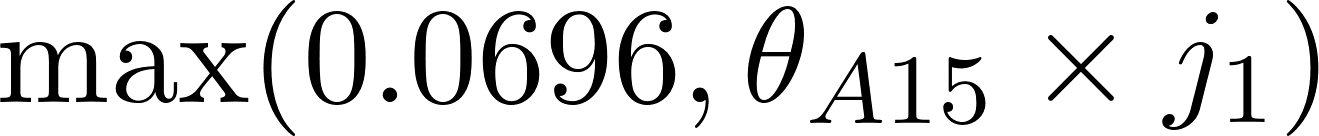](https://www.codecogs.com/eqnedit.php?latex=%5Cmax(0.0696%2C%20%5Ctheta_%7BA15%7D%20%5Ctimes%20j_1)#0) |
| [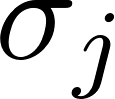](https://www.codecogs.com/eqnedit.php?latex=%5Csigma_j#0)  Rate per year of reactivation | [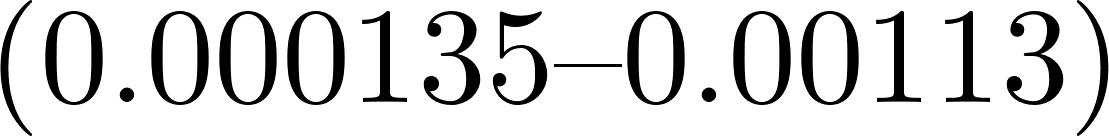](https://www.codecogs.com/eqnedit.php?latex=(0.000135%5Ctextendash0.00113)#0) | Retained if value for children was **less** than value for adults | Sample [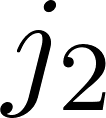](https://www.codecogs.com/eqnedit.php?latex=j_2#0)  from [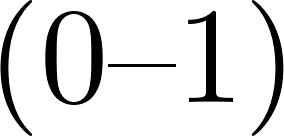](https://www.codecogs.com/eqnedit.php?latex=(0%5Ctextendash1)#0) | Sample [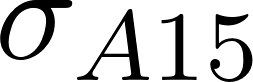](https://www.codecogs.com/eqnedit.php?latex=%5Csigma_%7BA15%7D#0) from  [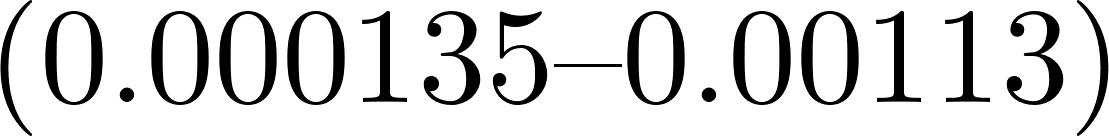](https://www.codecogs.com/eqnedit.php?latex=(0.000135%5Ctextendash0.00113)#0) | [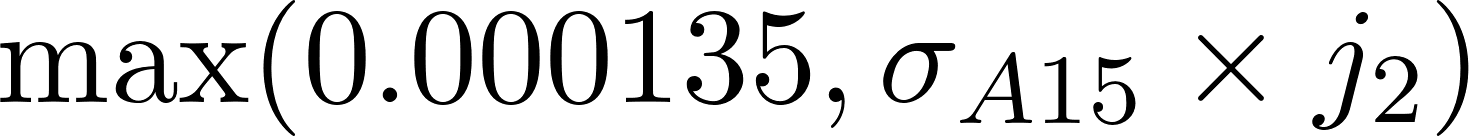](https://www.codecogs.com/eqnedit.php?latex=%5Cmax(0.000135%2C%20%5Csigma_%7BA15%7D%20%5Ctimes%20j_2)#0) |
| [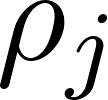](https://www.codecogs.com/eqnedit.php?latex=%5Crho_j#0)  Rate per year of relapse | [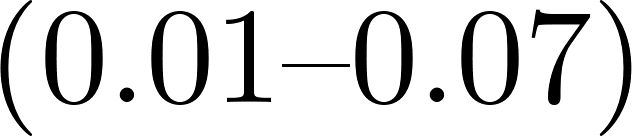](https://www.codecogs.com/eqnedit.php?latex=(0.01%5Ctextendash0.07)#0) | Retained if value for children was **less** than value for adults | Sample [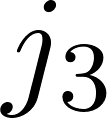](https://www.codecogs.com/eqnedit.php?latex=j_3#0)  from [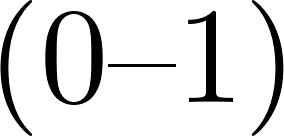](https://www.codecogs.com/eqnedit.php?latex=(0%5Ctextendash1)#0) | Sample [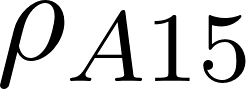](https://www.codecogs.com/eqnedit.php?latex=%5Crho_%7BA15%7D#0) from  [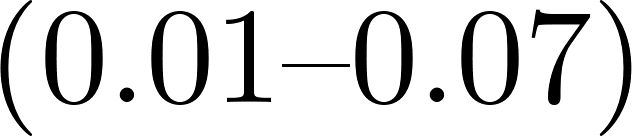](https://www.codecogs.com/eqnedit.php?latex=(0.01%5Ctextendash0.07)#0) | [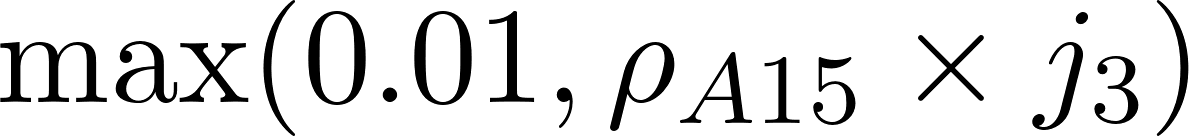](https://www.codecogs.com/eqnedit.php?latex=%5Cmax(0.01%2C%20%5Crho_%7BA15%7D%20%5Ctimes%20j_3)#0) |
| [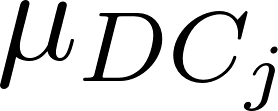](https://www.codecogs.com/eqnedit.php?latex=%5Cmu_%7BDC_j%7D#0)  Clinical TB mortality rate per year | [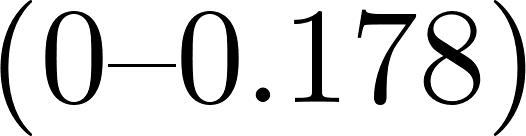](https://www.codecogs.com/eqnedit.php?latex=(0%5Ctextendash0.178)#0) | Retained if value for children was **greater** than value for adults | Sample [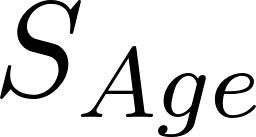](https://www.codecogs.com/eqnedit.php?latex=S_%7BAge%7D#0) from  [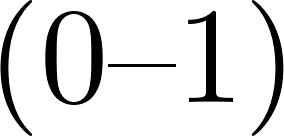](https://www.codecogs.com/eqnedit.php?latex=(0%5Ctextendash1)#0) | [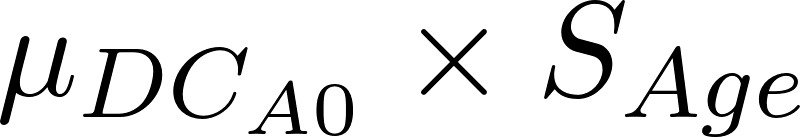](https://www.codecogs.com/eqnedit.php?latex=%5Cmu_%7BDC_%7BA0%7D%7D%20%5Ctimes%20S_%7BAge%7D#0) | Sample [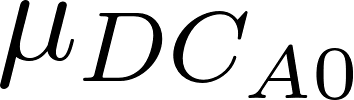](https://www.codecogs.com/eqnedit.php?latex=%5Cmu_%7BDC_%7BA0%7D%7D#0) from  [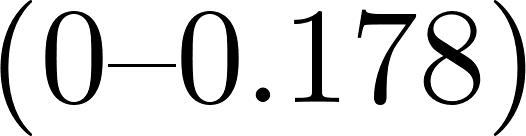](https://www.codecogs.com/eqnedit.php?latex=(0%20%5Ctextendash%200.178)#0) |
| [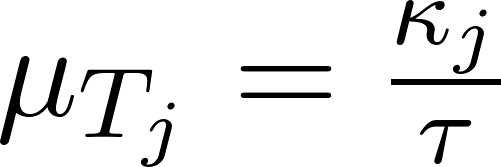](https://www.codecogs.com/eqnedit.php?latex=%5Cmu_%7BT_j%7D%20%3D%20%5Cfrac%7B%5Ckappa_j%7D%7B%5Ctau%7D#0)  On-treatment mortality rate per year | [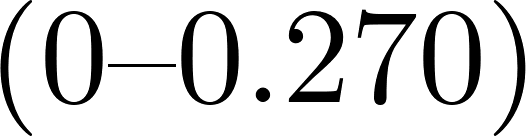](https://www.codecogs.com/eqnedit.php?latex=(0%5Ctextendash0.270)#0) | Retained if value for children was **greater** than value for adults | Sample [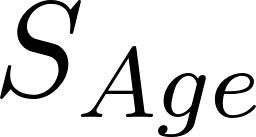](https://www.codecogs.com/eqnedit.php?latex=S_%7BAge%7D#0) from [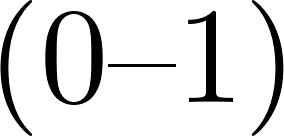](https://www.codecogs.com/eqnedit.php?latex=(0%5Ctextendash1)#0) | [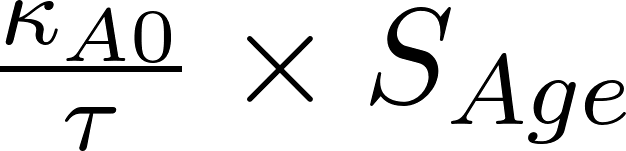](https://www.codecogs.com/eqnedit.php?latex=%20%5Cfrac%7B%5Ckappa_%7BA0%7D%7D%7B%5Ctau%7D%5Ctimes%20S_%7BAge%7D#0) | Sample [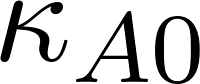](https://www.codecogs.com/eqnedit.php?latex=%5Ckappa_%7BA0%7D#0) from  [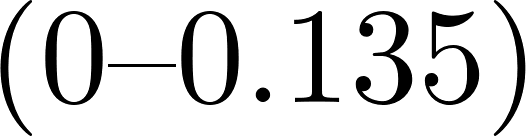](https://www.codecogs.com/eqnedit.php?latex=(0%5Ctextendash0.135)#0) |

### 1.3 TB treatment initiation and outcomes

Steps for calculating treatment initiation, treatment completion, non-completion, and mortality rates are described in the Additional File for Clark et. al., and included below with minor modification.^1^

TB treatment was assumed to start in 1960, aligned roughly with the discovery and widespread use of rifampicin, and increase following a sigmoid curve to 2019. The treatment initiation rate parameter, [
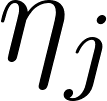
](https://latex-staging.easygenerator.com/eqneditor/editor.php?latex=%5Ceta_j#0), represents the age specific rate of treatment initiation from the clinical disease compartment to the on-treatment compartment. During calibration, a value for [
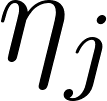
](https://latex-staging.easygenerator.com/eqneditor/editor.php?latex=%5Ceta_j#0) was sampled between 0 and 1. [
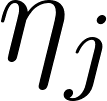
](https://latex-staging.easygenerator.com/eqneditor/editor.php?latex=%5Ceta_j#0) was multiplied by an age scaling parameter for children, [
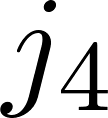
](https://latex-staging.easygenerator.com/eqneditor/editor.php?latex=j_4#0), also sampled between 0 and 1, to ensure that the treatment initiation rate in children was less than in adults. This was then multiplied by the value of the sigmoid curve at each year. The treatment initiation rate was calibrated to the notification rate in 2019 overall and by age reported by the World Health Organization (WHO).

We assumed the “SFR” is the ratio between treatment completions to the sum of treatment completions and non-completions. In India, [
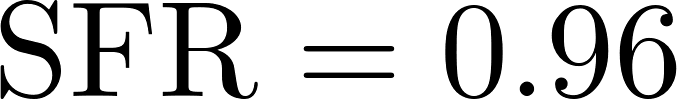
](https://www.codecogs.com/eqnedit.php?latex=%5Ctext%7BSFR%7D%20%3D%200.96#0). The data used to calculate the on-treatment outcomes was obtained from WHO. However, as the private sector accounts for a substantial portion of treatments in India, and not all the treatments conducted in the private sector are reported, we adjust the on-treatment completion and non-completion fractions from Table S3 as described below and in Table S4.

**Table S4** **Calculating treatment outcome parameter values for adults and children**

| **Parameter** | **Adults** | **Children** |
| --- | --- | --- |
| [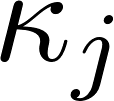](https://www.codecogs.com/eqnedit.php?latex=%5Ckappa_j#0)  On-treatment mortality fraction | [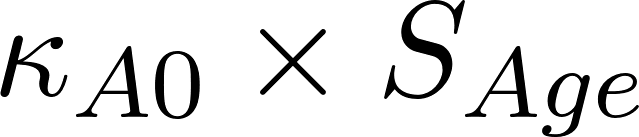](https://www.codecogs.com/eqnedit.php?latex=%5Ckappa_%7BA0%7D%20%5Ctimes%20S_%7BAge%7D#0) | Sample [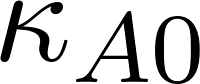](https://www.codecogs.com/eqnedit.php?latex=%5Ckappa_%7BA0%7D#0) from [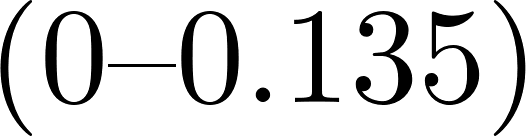](https://www.codecogs.com/eqnedit.php?latex=(0%5Ctextendash0.135)#0) |
| [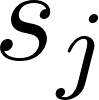](https://www.codecogs.com/eqnedit.php?latex=s_j#0)  On-treatment completion fraction | [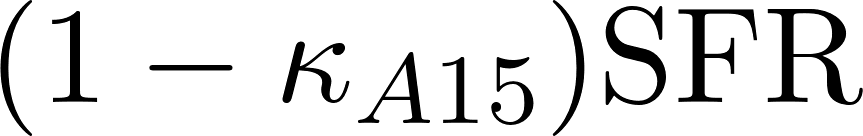](https://www.codecogs.com/eqnedit.php?latex=(1-%5Ckappa_%7BA15%7D)%5Ctext%7BSFR%7D#0) | [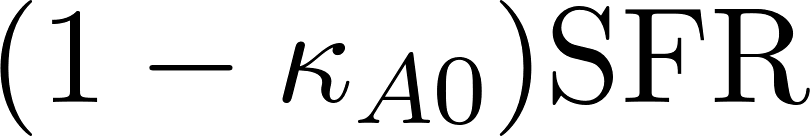](https://www.codecogs.com/eqnedit.php?latex=(1-%5Ckappa_%7BA0%7D)%5Ctext%7BSFR%7D#0) |
| [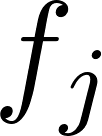](https://www.codecogs.com/eqnedit.php?latex=f_j#0)  On-treatment non-completion fraction | [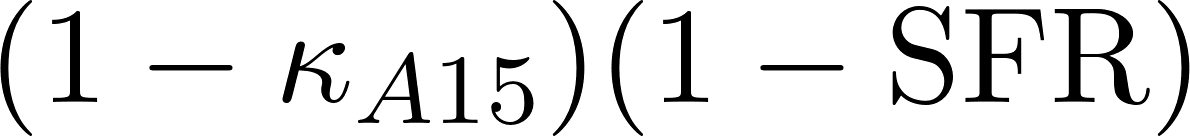](https://www.codecogs.com/eqnedit.php?latex=(1-%5Ckappa_%7BA15%7D)(1-%5Ctext%7BSFR%7D)#0) | [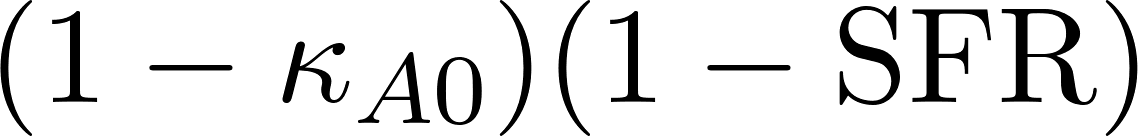](https://www.codecogs.com/eqnedit.php?latex=(1-%5Ckappa_%7BA0%7D)(1-%5Ctext%7BSFR%7D)#0) |

We assumed the total number of treatments is composed of the treatments that are reported and the treatments that are not reported. We assumed that the on-treatment mortality fraction is the same in the public and private sector but want to adjust the treatment completion and non-completion rates to account for differences between those reported and those not reported.

We assumed that 60% of the total treatment occurs in the public sector and the remaining 40% occurs in the private sector. We assumed that all treatments not reported are from the private sector, that the treatment completion rate in the private sector is 40%, and that there is no reporting bias (in that they were equally likely to not report treatment completions or non-completions or deaths). Before 2012, only the treatment conducted in the public sector was reported, but since then, treatment in the private sector has begun to be reported.

**Table S5 Calculation of treatment outcomes for India by year**

| **Description** | **Symbol** | [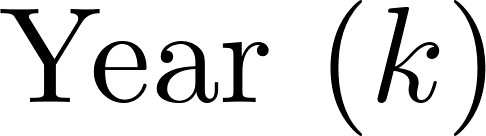](https://www.codecogs.com/eqnedit.php?latex=%5Ctext%7BYear%20%7D%20(k)#0) | | | | | | | | |
| --- | --- | --- | --- | --- | --- | --- | --- | --- | --- | --- |
|  |  | **≤2012** | **2013** | **2014** | **2015** | **2016** | **2017** | **2018** | **2019** | **≥2020** |
| Fraction of total treatments reported | [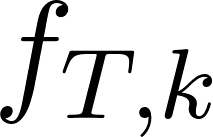](https://www.codecogs.com/eqnedit.php?latex=f_%7BT%2Ck%7D#0) | 0.60 | 0.63 | 0.68 | 0.67 | 0.73 | 0.77 | 0.80 | 0.83 | 0.87 |
| On-treatment mortality rate | [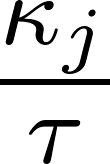](https://www.codecogs.com/eqnedit.php?latex=%5Cfrac%7B%5Ckappa_j%7D%7B%5Ctau%7D#0) | [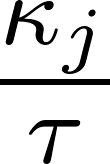](https://www.codecogs.com/eqnedit.php?latex=%5Cfrac%7B%5Ckappa_j%7D%7B%5Ctau%7D#0)  Sample  from , then | | | | | | | | |
| On-treatment completion rate |  |  | | | | | | | | |
| On-treatment non-completion rate |  |  | | | | | | | | |

### 1.4 Incorporating BMI into the TB model

***BMI strata***

We defined four body mass index (BMI) strata to include as an additional dimension in the TB transmission model: moderate to severe thinness (BMI < 17.0 kg/m^2^), mild thinness (17.0 kg/m^2^ ≤ BMI < 18.5 kg/m^2^), normal BMI (18.5 kg/m^2^ ≤ BMI < 25.0kg/m^2^) and overweight to obese BMI (BMI ≥ 25.0 kg/m^2^).

***Distribution of the population in each BMI strata***

The model was designed to align with the proportion of the population within each BMI category for each age and year supplied by external BMI distribution files. The age-standardised proportion of the adult population >19 years within each strata was taken from the Global Health Observatory for the years 1975–2016.[^19^](https://www.zotero.org/google-docs/?NcRoOZ) This consisted of the proportion of the population overweight (BMI ≥ 25.0 kg/m^2^) or thin (BMI < 18.5 kg/m^2^), from which the proportion of normal weight was calculated. The proportion thin was separated into moderate to severe thinness and mild thinness based on trends in the ratio of each in the India Demographic and Health Surveys from 2005–2006, 2015–2016 and 2019–2021,[^20–22^](https://www.zotero.org/google-docs/?LoExJ5) weighted by the male:female ratio from United Nations (UN) population estimates[^3^](https://www.zotero.org/google-docs/?kEbf2Q) and using the survey package in R.[^23^](https://www.zotero.org/google-docs/?KwvFIR)

To estimate the proportion of children and adolescents in each BMI strata, we again used the Global Health Observatory.[^19^](https://www.zotero.org/google-docs/?TlCp8V) We separated children aged 0–4 years into BMI strata based on standard deviations (SD) for weight-for-height of severe wasting (weight-for-height < -3SD), wasting (-3SD ≤ weight-for-height < -2SD), normal (-2SD ≤ weight-for-height < +2SD) and overweight (weight-for-height ≥ +2SD), aligning with our four BMI strata. We note that estimates were only available for normal, wasting and severe wasting for the years 1999, 2006, 2014, 2015 and 2017, while for overweight these were available for 1994–2019.

We separated children and adolescents aged 5–19 years also based on SD, but in this instance for BMI, of thinness (BMI < -2SD), normal (-2SD ≤ BMI < +1SD) and overweight (BMI ≥ +2SD). These estimates were available for 1975–2016. The proportion thin was separated as for adults, into moderate to severe thinness (BMI < -3SD) and mild thinness (-3SD ≤ BMI < -2SD) based on trends in the ratio of each in adults in Demographic and Health Surveys.

***Varying parameters by BMI strata***

We re-estimated the relationship between BMI and TB incidence from Lönnroth et al,^24^ using updated data from Cegielski et al,^25^ and assuming a uniform distribution for BMI within each BMI strata. We used this to fit a log-linear model with a dependent variable of TB incidence, an independent variable of BMI, and a categorical variable for the studies reported in Lönnroth et al.^24^

Using the log-linear model, the BMI distributions from above, and estimated population-level TB incidence in 2015 (TB incidence in 2019–2021 may have been potentially affected by COVID-19-associated disruptions, while 2005–2006 did not include primary sampling units in IPUMS and 1998–1999 did not include data for men),^20–22^ we estimated the expected TB incidence for each BMI strata weighted by the female:male ratio. We used this to calculate the risk ratio for TB in each stratum compared to the stratum with normal BMI, assuming that differences in risk of incident TB were a direct result of differences in rate of progression or reversion to TB disease. We estimated the mean and uncertainty intervals for this from 10,000 runs of the process (Table S6).

Treatment outcome estimates were calculated using data collected from adults with microbiologically confirmed TB in five sites of the Regional Prospective Observational Research on Tuberculosis (RePORT) India consortium from 2015–2019.^26^ The primary exposure was BMI at treatment initiation and the outcome was a composite of death, treatment failure, and relapse/recurrence. We conducted multivariable analysis using Poisson regression, in which we used person-time as an offset to calculate adjusted incidence rate ratios (aIRR). We calculated person-time from the time of treatment initiation to the occurrence of the first mutually exclusive outcome of interest (failure/recurrence/death), loss to follow-up, or until right-censoring at 24 months of follow-up. In multivariable analysis, we included age, sex, and cough duration in the multivariable a priori and included other potential confounders based on prior literature that were significant at the p<0.2 level. The original publication calculated risk for different BMI categories than the ones required for this model. We recalculated the aIRR for the following BMI categories: moderately to severely thin (BMI < 17.0 kg/m^2^), mildly thin (17.0 kg/m^2^ ≤ BMI < 18.5 kg/m^2^), normal (18.5 kg/m^2^ ≤ BMI < 25.0kg/m^2^) and overweight to obese (BMI ≥ 25.0 kg/m^2^).

We assumed that the risk ratios for disease progression, reversion, and treatment outcomes were different by BMI strata, and were applied identically to adults, adolescents, and children.

**Table S6** Risk ratios for natural history and treatment outcome parameters by BMI strata

| **BMI strata** | **BMI interval** | **Risk ratio**  **[95% CI] for progression & reactivation parameters**  **θ_j_, σ_j_, ⍴_j_** | **Risk ratio**  **[95% CI] for treatment non-**  **completion parameter**  **f_j_** | **Risk ratio**  **[95% CI] for on-treatment mortality parameter**  **μ_Tj_** |
| --- | --- | --- | --- | --- |
| Moderate to severe thinness | <17.0 kg/m^2^ | 1.73  (1.57, 1.89) | 1.58  (1.09, 2.31) | 3.38  (1.84, 6.48) |
| Mild thinness | 17.0–18.4 kg/m^2^ | 1.43  (1.32, 1.54) | 1.14  (0.72, 1.79) | 0.75  (0.24, 1.93) |
| Normal BMI | 18.5–24.9 kg/m^2^ | Reference | Reference | Reference |
| Overweight to obese BMI | >25.0 kg/m^2^ | 0.26  (0.21, 0.31) | 0.82  (0.34, 1.69) | 1.14  (0.26, 3.47) |

###

### 1.5 TB model simulation, calibration, and validation

We followed the same process for model simulation and calibration as in Clark et al.,[^1^](https://www.zotero.org/google-docs/?rXIiDO) reproduced here with minor modifications.

We specified a system of ordinary differential equations defining the derivatives with respect to time of a set of state variables, to simulate the country-specific tuberculosis epidemic between 1900 and 2050. We initialised the simulation by distributing the population between the eight tuberculosis natural history states using a fitted parameter representing the proportion of the population uninfected at the start of the simulation. For each year of the simulation (1900–2050), our models are designed to exactly match the age and country-specific UN population estimates and projections.

Broadly, the modelling approach was as follows:

1. Construct a mechanistic model
2. Calibrate the model by identifying areas of the input parameter space where the output of the mechanistic model was consistent with the historical epidemiologic data
3. Use the calibrated model to simulate and predict future tuberculosis epidemiology and model interventions

In the context of this analysis, step 1 was achieved by creating the compartment differential equation model as specified in Section 1. For step 2, we independently calibrated a model by identifying areas of the parameter space (defined by prior ranges for TB natural history parameters [Table S1] and parameters linking BMI to the risk of TB progression and outcomes [Table S6]) that made the output of the model match the corresponding calibration targets (Table S7 below). The model was fitted to the calibration targets using history matching with emulation, a method that allowed us to explore high-dimensional parameter spaces efficiently and robustly.^27–30^ History matching progressed as a series of iterations, called waves, where implausible areas of the parameter space, i.e., areas that were unable to give a match between the model output (e.g., the predicted incidence rate by the model) and the empirical data (e.g., the incidence rate calibration target from WHO data), were found and discarded. These implausible areas are discarded as they are not consistent with the empirical data, i.e the model is unable to fit the observed data using parameters from this space. Therefore, finding a solution to these areas will never be possible, given the constraints from the model structure and the observed data. This is unlikely to lead to overfitting and leads to more comprehensive estimates of the true uncertainty in the results than other calibration methods. In order to identify implausible parameter sets, emulators, which are statistical approximations of model outputs that are built using a modest number of model runs, were used. Emulators provide an estimate of the value of the model at any parameter set of interest, with the advantage that they are orders of magnitude faster than the model.

History matching with emulation, implemented through the *hmer* package in R,^31,32^ considerably reduced the size of the parameter space to investigate. Rejection sampling was then performed on the reduced space to identify at least 1000 parameter sets that matched all targets. If we were unable to find at least 1000 fully fitted parameter sets using history matching with emulation, we subsequently used an Approximate Bayesian Computation using Markov Chain Monte Carlo method (ABC-MCMC). ABC-MCMC was conducted using the *easyABC* package in R, modified by Sebastian Funk, Gwenan Knight, and the Tuberculosis Modelling group at LSHTM for adaptive sampling and to accept seeded parameter values.[^33,34^](https://www.zotero.org/google-docs/?BiMMSj) We used parameter sets with the maximum number of targets fitted using history matching with emulation as starting seeds for multiple MCMC chains, with the ABC-MCMC algorithm continuously adapting using the last 1000 points, a burn in of 1000 samples, and the noise factor set to 0.0001.

Once we had obtained 1000 parameter sets that produced output consistent with the calibration targets, we used those parameter sets with the mechanistic model to simulate the future while representing the uncertainty associated with TB natural history parameters and links between BMI and TB (step 3). We assumed that current trends and quality of tuberculosis services continued into the future at the same rate.

**Table S7** TB model calibration targets for India

| **Calibration Targets** | **Year** | **Age (years)** | **Estimate** | **Lower** | **Upper** |
| --- | --- | --- | --- | --- | --- |
| Tuberculosis incidence rate (per 100,000 population/year) | 2000^3,7^ | All | 289 | 149 | 473 |
|  | 2020^3,7,35^ | All | 188 | 129 | 257 |
|  |  | 0–14 | 91 | 56 | 126 |
|  |  | ≥15 | 224 | 138 | 310 |
| Tuberculosis mortality rate (per 100,000 population/year) | 2000^3,7^ | All | 67 | 57 | 79 |
|  | 2020^3,7^ | All | 37 | 34 | 40 |
| Tuberculosis case notification rate  (per 100,000 population/year) | 2000^3,8^ | All | 177 | 142 | 212 |
|  | 2020^3,8^ | All | 136 | 109 | 163 |
|  |  | 0–14 | 33 | 26 | 40 |
|  |  | ≥15 | 173 | 138 | 208 |
| Active tuberculosis prevalence  (per 100,000 population) | 2015^36^ | All | 315 | 210 | 529 |
|  | 2021^37^ | All | 312 | 218 | 406 |
|  | 2021[^3^](https://www.zotero.org/google-docs/?ZFWnqu)^7^ | ≥15 | 394 | 276 | 512 |
| Tuberculosis infection  prevalence proportion | 2021^37^ | All | 0.314 | 0.114 | 0.514 |
| Subclinical tuberculosis  prevalence ratio | 2020^38^ | All | 0.504 | 0.361 | 0.797 |

We validated our model outputs by comparing to estimates of the population attributable fraction (PAF) for undernutrition. The PAF was calculated as:

Where  represented the incidence of disease in those with moderate to severe thinness and mild thinness,  represented the incidence of disease in those with normal or overweight BMI,  was the number of TB cases in those with moderate to severe thinness and mild thinness, and  was the number of TB cases overall.

We also used self-reported TB status by BMI strata from the India 2015–2016 Demographic and Health Survey as a validation.[^21^](https://www.zotero.org/google-docs/?qCK9BC)

## Climate and food systems model

### 2.1 LandSyMM

The Land System Modular Model (LandSyMM), is a state of the art global land use model that integrates a dynamic global vegetation model (LPJ-GUESS)^39^ with a land system model (PLUM)^40^ and a Modified, Implicit, Directly Additive Demand System (MAIDADS).^41^ More information about LandSyMM, LPJ-GUESS, PLUM and MAIDADS can be found from <https://landsymm.earth> and described briefly below. LandSyMM combines spatially-explicit, biophysically-derived yield responses with socio-economic scenario data to project future demand, land use, and land management inputs. Here, LandSyMM used climate input data from the fifth Coupled Model Intercomparison Project (CMIP5)[^42^](https://www.zotero.org/google-docs/?0WmM2w) for the IPSL-CM5A-MR climate model.[^43^](https://www.zotero.org/google-docs/?WlYzoU) LandSyMM improves upon existing integrated assessment models (IAMs) by (a) modelling crop yield responses in a more detailed manner at a finer grain, and (b) calculating commodity demand endogenously and therefore, unlike most land use models, demand for commodities responds dynamically to changing commodity prices.

MAIDADS^41^ uses per-capita income levels, food prices and price elasticities to estimate subsistence and discretionary consumption levels and captures nonlinear relationships between food demand and income. As incomes rise, consumption shifts away from staple foods (cereals, oil crops and pulses) towards greater consumption of meat and fruit and vegetables. Conversely, as prices increase, overall consumption decreases and shifts away from ‘luxury’ goods such as meat, fruit and vegetables back towards staple crops. If subsistence levels of consumption are too expensive for a country, then demand for food products is calculated by scaling desired subsistence consumption by the ratio between income available for food expenditure and the desired subsistence consumption.

Increasing demand for commodities is met by in-country expansion or intensification of crop production or by imports from the global market. Excess commodity production in a country is exported to the global market. Bilateral trading is not currently modelled in PLUM. LandSyMM reflects reality in agricultural markets where supply adjusts slowly and therefore will not always meet demand in a particular year. The global market is not constrained to be in equilibrium, instead allowing over- or under-supply of commodities buffered through global stocks. Global market prices are adjusted on an annual basis based on the net balance of imports and exports. For example, oversupply of a commodity on the global market decreases the price as stocks rise, this reduces the benefits from its export and reduces the cost of importing it. The restrictions to exports from Russia and Ukraine were applied exogenously as a constraint on the maximum level of exports for each commodity, calculated from the supply in 2021 and a percentage reduction in exports.

### 2.2 Population weight distributions

We calculated the proportion of the population that was underweight (BMI < 18.5 kg/m^2^), normal weight (BMI 18.5–25 kg/m^2^), overweight (BMI 25–30 kg/m^2^) or obese (BMI > 30.0 kg/m^2^) globally and in India for a given year by estimating the mean BMI to use as input in a log normal distribution.[^44^](https://www.zotero.org/google-docs/?6mw2kU)

We estimated the mean BMI of a country’s population using the following relationship:

*(1)*

Where  was a country fixed effect,  was the average calorie consumption per person per day in a country,  was the percentage of daily calories consumed in the form of animal products in a country, and  represented the error term. The relationship in *(1)* was estimated by regressing food consumption data from The Food and Agriculture Organization Corporate Statistical Database with WHO estimates of mean BMI for the years 2000–2017 (R^2^ = 0.87).

We used the estimated mean BMI of a country to calculate the different population weight proportions for a given time step according to a log normal distribution with a mean:

*(2)*

and standard deviation:

*(3)*

Where  was constant over time and calculated by fitting a log-normal distribution to WHO estimates of mean BMI and the prevalence of underweight, overweight and obesity in 2010 using a cross-entropy method. The cross-entropy approach estimates the parameters of the log-normal distribution by comparing two probability distributions and minimising the Kullback-Leibler Divergence. We estimated moderate to severe thinness (as a proportion of all thinness) by assuming that trends in this identified above from the India Health and Demographic Survey continued into the future.

## Future scenarios

We modelled four scenarios from Alexander *et al*.[^45^](https://www.zotero.org/google-docs/?4bfga8):

1. *No Shocks*
2. *Export Restriction Shock*
3. *Energy Price Shock*
4. *Export and Energy Shocks*

The scenarios explored the siloed and combined effects of (a) export restrictions from Ukraine and Russia and (b) higher agricultural input costs associated with higher energy prices. The socio-economic and climate basis for the four scenarios was identical; all scenarios used country gross domestic product (GDP) and populations projections for the ‘Middle of the Road’ Shared Socioeconomic Pathway (SSP2)[^46^](https://www.zotero.org/google-docs/?x1KZsY) and climate change projections based on Representative Concentration Pathway (RCP) 4.5.[^47^](https://www.zotero.org/google-docs/?MgQaq5) A Monte Carlo approach to explore uncertainty associated with input parameters was used and parameters were sampled from LandSyMM using a Sobol sequence method with 30 ensemble members, with further details in the Additional File for Alexander et al.^45^

The Russia and Ukraine food export restrictions represented the imposition of strong sanctions against Russian exports, assuming that between 75% and 100% of the export levels from 2021 were unable to continue from 2022 onwards. The range was sampled as a uniform distribution for the ensemble members. Energy price shocks in the relevant scenarios were implemented by three parameter changes, relating to fertiliser costs, management intensity costs and food transport costs. In the absence of an energy shock the costs remained constant, at the sampled parameter value, throughout the simulation. In scenarios with an energy shock, fertiliser prices were increased by 200% and management intensity and transport costs were increased by 50% and remained at this level until 2040. These price increases were implemented as a scaling of the LandSyMM parameter probability distributions.

We assumed the quality and coverage of current TB interventions would remain constant after 2019 until the end of the simulations in 2040. Simulating until 2040 was a stylised approach that provided insight into the short-term implications, as well as a better understanding of how the global system could be reconfigured over a longer time period, and the effect of that on TB.

# ADDITIONAL RESULTS

## Epidemiological trends in the *No Shocks* scenario

Trends in tuberculosis epidemiology from 1980–2040 for the *No Shocks* scenario are shown in Figure S2.

**Figure S2** Model simulated baseline trends in epidemiology

***Trends in BMI in the No Shocks scenario***

In the *No Shocks* scenario, 12.5% (95% uncertainty interval = 11.8, 13.4) and 8.5% (8.1, 9.1) of the population in 2022 overall was predicted to have mild and moderate thinness respectively, compared to 18.1% (17.3, 19.2) and 17.9% (17.0, 18.7) among people with TB. In 2035, the proportion of the population with mild thinness and moderate to severe thinness was predicted to decrease to 10.0% (9.5, 10.6) and 6.3% (6.0, 6.7) respectively, with corresponding declines in the proportion of mild and moderate to severe thinness among people with TB (16% (15.3, 16.8) and 14.8% (14.2, 15.5) respectively). In the *No Shocks* scenario in 2022, 16.4% (15.2, 17.5) of the population was predicted to have an overweight BMI, compared to 5.1% (4.1, 6) among people with TB. The proportion of the population with an overweight BMI was predicted to increase in 2035, with 21.8% (20.8, 22.7) of the overall population, and 6.8% (6.3, 7.3) among people with TB.

***Validation with PAF and self-reported TB incidence by BMI strata***

Estimates of the PAF for undernutrition for TB are provided in Table S8, and comparisons between the model output TB incidence by BMI strata for all ages and adults and the self-reported TB incidence by BMI strata from the India 2015–2016 Demographic and Health Survey are in Table S9. The model predicted PAF was 18.4% (95% uncertainty interval = 17.8, 19.1), which falls on the lower end of the range of the revised and updated estimate from Bhargava et al., 2022 (45.2% [17.0, 71.0]). Similarly, the model predicted a lower proportion of TB in the lower BMI strata compared to estimates (Table S9). Whereas the self-reported incidence of TB in those with moderate to severe thinness was 1,093 (956, 1 230) per 100,000 population, the model predicted incidence for adults was almost 2.5 times lower.

As noted in section 1.4, there was limited data available to capture the relationship between low BMI and TB. The model was calibrated to reported estimates of incidence overall, using risk ratios from Table S6, where the maximum for the increase in progression to TB disease for moderate to severe thinness is less than 2 times the rate for those with normal BMI. The low rate ratios may have resulted in an underestimation of the amount of TB that occurred in the two low BMI strata. Additionally, individuals are assumed to mix randomly in the model, which may not be the case, and it is likely that assortative mixing would contribute to increasing burden of TB in lower BMI strata.

**Table S8** Estimates of the population attributable fraction of undernutrition for India

| **Study and measure** | **PAF for India**  **(95% CI)** |
| --- | --- |
| WHO estimates, 2023 | 26.4% (22.3, 30.8) |
| Bhargava., 2022  (Using prevalence of undernourishment and RR = 3.2) | 24.8% |
| Bhargava., 2022  (Using prevalence of undernutrition and original RR = 3.2 | 34.2% (28.6, 39.3) |
| Bhargava., 2022  (Using prevalence of undernutrition and revised RR = 4.49 (2.28, 8.86)) | 45.2% (17.0, 71.0) |
| Model predicted PAF | 18.4% (17.8, 19.1) |

**Table S9** Self-reported TB incidence by BMI strata

| **BMI strata** | **BMI interval** | **Self-reported TB in 2015 per 100,000 [95% CI]** | **Model TB in 2015 per 100,000 for all ages [95% UI]** | **Model TB in 2015 per 100,000 for adults [95% UI]** |
| --- | --- | --- | --- | --- |
| Moderate to severe thinness | <17.0 kg/m^2^ | 1 093  (956, 1 230) | 375.6  (342.8, 406.2) | 441.9  (399.9, 484.8) |
| Mild thinness | 17.0–18.4 kg/m^2^ | 431  (367, 496) | 273.7  (254.6, 293.9) | 325.3  (299.6, 355.4) |
| Normal BMI | 18.5–24.9 kg/m^2^ | 234 (209, 260) | 185.3  (172.3, 199) | 222  (205.7, 241.8) |
| Overweight to obese BMI | ≥25.0 kg/m^2^ | 145  (11, 181) | 53.3  (47.7, 61.2) | 55.5 (49.4, 64.1) |

##

##

## Future scenario results

Trends in the TB incidence and TB mortality rates for the *No Shocks* and three future scenarios are shown in Figure S3.

**Figure S3** Trends in TB incidence and mortality rates for all scenarios

***Trends in BMI in the future scenarios***

With the *Export Restriction Shock* scenario, the proportion of the population in 2035 with moderate to severe thinness, mild thinness, normal BMI, and overweight BMI was 6.4% (6.1, 6.8), 10.0% (9.6, 10.7), 61.9% (61.7, 62.0), and 21.7% (20.5, 22.6) respectively (Table S10). With the *Energy Price Shock* scenario, the proportion of the population in 2035 with moderate to severe thinness, mild thinness, normal, and overweight BMI was 6.9% (6.6, 7.5), 10.9% (10.4, 11.7), 62.0% (62.0, 62.1), and 20.2% (18.8, 21.0) respectively (Table S10). With the *Export and Energy Shocks* scenario, the changes to BMI increased further, and the proportion of the population in 2035 with moderate to severe thinness, mild thinness, normal BMI, and overweight BMI was 7.0% (6.6, 7.5), 11.0% (10.4, 11.7), 62.0% (62.0, 62.1), and 20.0% (18.8, 21.0) respectively (Table S10). Table S11 and S12 provide the proportion of the population in each BMI strata for ages 0–14 and ages 15+ respectively.

**Table S10** BMI distribution in 2022 (*No Shocks* scenario only) and 2035 (all scenarios) among those with TB disease and the population overall

| **Year** | **Scenario** | **BMI strata** | **Among people with  TB, % (95% UI)** | **Among population overall, % (95% UI)** |
| --- | --- | --- | --- | --- |
| 2022 | *No Shocks* | Overweight BMI | 5.1%  (4.1, 6.0) | 16.4%  (15.2, 17.5) |
|  |  | Normal BMI | 58.9%  (58.0, 59.6) | 62.6%  (62.2, 62.7) |
|  |  | Mild thinness | 18.1%  (17.3, 19.2) | 12.5%  (11.8, 13.4) |
|  |  | Moderate to severe thinness | 17.9%  (17.0, 18.7) | 8.5%  (8.1, 9.1) |
| 2035 | *No Shocks* | Overweight BMI | 6.8%  (6.3, 7.3) | 21.8%  (20.7, 22.7) |
|  |  | Normal BMI | 62.4%  (61.6, 63.2) | 61.9%  (61.7, 62.0) |
|  |  | Mild thinness | 16.0%  (15.3, 16.8) | 10.0%  (9.5, 10.6) |
|  |  | Moderate to severe thinness | 14.8%  (14.2, 15.5) | 6.3%  (6.0, 6.7) |
|  | *Export  Shocks* | Overweight BMI | 6.8% (6.2, 7.3) | 21.7%  (20.5, 22.6) |
|  |  | Normal BMI | 62.4% (61.5, 63.1) | 61.9%  (61.7, 62.0) |
|  |  | Mild thinness | 16.0%  (15.3, 16.9) | 10.0%  (9.6, 10.7) |
|  |  | Moderate to severe thinness | 14.9% (14.2, 15.5) | 6.4%  (6.1, 6.8) |
|  | *Energy  Shocks* | Overweight BMI | 6.1% (5.5, 6.6) | 20.2%  (18.8, 21.0) |
|  |  | Normal BMI | 61.3% (60.3, 62.1) | 62.0%  (62.0, 62.1) |
|  |  | Mild thinness | 16.9% (16.2, 17.9) | 10.9%  (10.4, 11.7) |
|  |  | Moderate to severe thinness | 15.6% (15.0, 16.4) | 6.9%  (6.6, 7.5) |
|  | *Export &  Energy  Shocks* | Overweight BMI | 6.1% (5.6, 6.6) | 20.0%  (18.8, 21.0) |
|  |  | Normal BMI | 61.3% (60.3, 62.1) | 62.0%  (62.0, 62.1) |
|  |  | Mild thinness | 17.0% (16.2, 17.9) | 11.0%  (10.4, 11.7) |
|  |  | Moderate to severe thinness | 15.7% (15.0, 16.4) | 7.0%  (6.6, 7.5) |

**Table S11** BMI distribution in 2022 (*No Shocks* scenario only) and 2035 (all scenarios) among those with TB disease and the general population aged 0–14

| **Year** | **Scenario** | **BMI strata** | **Among people with  TB, % (95% UI)** | **Among population overall, % (95% UI)** |
| --- | --- | --- | --- | --- |
| 2022 | *No Shocks* | Overweight BMI | 2.3%  (1.8, 2.7) | 6.1%  (5.6, 6.5) |
|  |  | Normal BMI | 65.1%  (63.8, 65.9) | 72.5%  (71.7, 73.1) |
|  |  | Mild thinness | 17.2%  (16.5, 18.2) | 13.2%  (12.6, 14.1) |
|  |  | Moderate to severe thinness | 15.4%  (14.9, 16.1) | 8.1%  (7.7, 8.6) |
| 2035 | *No Shocks* | Overweight BMI | 3.1%  (2.8, 3.3) | 8.1%  (7.6, 8.5) |
|  |  | Normal BMI | 69.8%  (68.8, 70.5) | 74.7%  (74.3, 74.9) |
|  |  | Mild thinness | 14.8%  (14.3, 15.5) | 11%  (10.5, 11.5) |
|  |  | Moderate to severe thinness | 12.3%  (11.9, 12.9) | 6.3%  (6.1, 6.6) |
|  | *Export  Shocks* | Overweight BMI | 3.1%  (2.8, 3.3) | 8.1%  (7.5, 8.4) |
|  |  | Normal BMI | 69.8%  (68.7, 70.4) | 74.6%  (74.2, 74.9) |
|  |  | Mild thinness | 14.8%  (14.4, 15.6) | 11%  (10.6, 11.6) |
|  |  | Moderate to severe thinness | 12.3%  (11.9, 12.9) | 6.3%  (6.1, 6.6) |
|  | *Energy  Shocks* | Overweight BMI | 2.8%  (2.5, 3) | 7.3%  (6.8, 7.7) |
|  |  | Normal BMI | 68.3%  (67.1, 69.2) | 74%  (73.4, 74.5) |
|  |  | Mild thinness | 15.8%  (15.3, 16.6) | 11.8%  (11.4, 12.6) |
|  |  | Moderate to severe thinness | 13.1%  (12.6, 13.8) | 6.8%  (6.5, 7.2) |
|  | *Export &  Energy  Shocks* | Overweight BMI | 2.8%  (2.5, 3) | 7.3%  (6.8, 7.7) |
|  |  | Normal BMI | 68.3%  (67.1, 69.2) | 74%  (73.4, 74.5) |
|  |  | Mild thinness | 15.9%  (15.3, 16.6) | 11.9%  (11.4, 12.6) |
|  |  | Moderate to severe thinness | 13.2%  (12.6, 13.8) | 6.8%  (6.5, 7.2) |

**Table S12** BMI distribution in 2022 (*No Shocks* scenario only) and 2035 (all scenarios) among those with TB disease and the general population aged 15+

| **Year** | **Scenario** | **BMI strata** | **Among people with  TB, % (95% UI)** | **Among population overall, % (95% UI)** |
| --- | --- | --- | --- | --- |
| 2022 | *No Shocks* | Overweight BMI | 5.7%  (4.6, 6.6) | 19.9%  (18.5, 21.2) |
|  |  | Normal BMI | 57.7%  (56.9, 58.3) | 59.2%  (59, 59.3) |
|  |  | Mild thinness | 18.3%  (17.4, 19.5) | 12.2%  (11.5, 13.2) |
|  |  | Moderate to severe thinness | 18.3%  (17.4, 19.2) | 8.7%  (8.2, 9.3) |
| 2035 | *No Shocks* | Overweight BMI | 7.4%  (6.9, 8) | 25.7%  (24.4, 26.7) |
|  |  | Normal BMI | 61.2%  (60.4, 61.8) | 58.3%  (58, 58.6) |
|  |  | Mild thinness | 16.1%  (15.4, 17.1) | 9.7%  (9.2, 10.3) |
|  |  | Moderate to severe thinness | 15.3%  (14.5, 15.9) | 6.3%  (6, 6.7) |
|  | *Export  Shocks* | Overweight BMI | 7.4%  (6.8, 7.9) | 25.5%  (24.2, 26.5) |
|  |  | Normal BMI | 61.2%  (60.4, 61.8) | 58.3%  (58, 58.6) |
|  |  | Mild thinness | 16.2%  (15.5, 17.1) | 9.7%  (9.3, 10.4) |
|  |  | Moderate to severe thinness | 15.3%  (14.5, 15.9) | 6.4%  (6.1, 6.8) |
|  | *Energy  Shocks* | Overweight BMI | 6.7%  (6, 7.2) | 23.7%  (22.2, 24.8) |
|  |  | Normal BMI | 60.2%  (59.2, 60.9) | 58.7%  (58.5, 58.9) |
|  |  | Mild thinness | 17.1%  (16.4, 18.1) | 10.6%  (10.1, 11.4) |
|  |  | Moderate to severe thinness | 16.1%  (15.3, 16.8) | 6.9%  (6.6, 7.5) |
|  | *Export &  Energy  Shocks* | Overweight BMI | 6.6%  (6.1, 7.1) | 23.6%  (22.2, 24.8) |
|  |  | Normal BMI | 60.2%  (59.2, 60.9) | 58.7%  (58.5, 58.9) |
|  |  | Mild thinness | 17.1%  (16.4, 18.1) | 10.7%  (10.1, 11.4) |
|  |  | Moderate to severe thinness | 16.1%  (15.3, 16.8) | 7.0%  (6.6, 7.5) |

***Impact on TB cases and deaths by BMI***

The TB incidence rate and number of TB cases each year between 2015 and 2040 for each of the BMI strata and scenarios are shown in Figure S4 and Figure S5.

Between 2022 and 2035, compared to the *No Shocks* scenario, the *Export Restriction Shock* scenario predicted 14.4 thousand (1.5, 27.6) more cases of TB with moderate to severe thinness, and 15.3 thousand (-0.7, 33.6) more cases with mild thinness. 4.7 thousand (0.2, 7.8) fewer cases were predicted to occur in those with overweight BMI.

Between 2022 and 2035, compared to the *No Shocks* scenario, the *Energy Price Shock* scenario predicted 412.8 thousand (328.7, 502.4) more TB cases and 92.4 thousand (73.3, 112.9) more TB deaths with moderate to severe thinness, and 465.0 thousand (373.8, 563.6) more TB cases and 73.9 thousand (59.0, 89.3) more TB deaths with mild thinness. 117.7 thousand (93.7, 143.9) fewer TB cases and 34.1 thousand (27.5, 41.9) fewer TB deaths were predicted to occur in those with overweight BMI.

Between 2022 and 2035, compared to the *No Shocks* scenario, the *Export and Energy Shocks* scenario predicted 432.0 thousand (351.3, 552.2) more TB cases and 96.8 thousand (77.8, 124.4) more TB deaths with moderate to severe thinness, and 489.2 thousand (398.3, 617.7) more TB cases and 77.4 thousand (63.3, 98) more TB deaths with mild thinness. 123.7 thousand (99.3, 158.4) fewer TB cases and 35.6 thousand (29.3, 44.8) fewer TB deaths were predicted to occur in those with overweight BMI.

**Figure S4** TB incidence rate over time by BMI and scenario

*Note: different scales for each BMI strata*

**Figure S5** Number of TB cases over time by BMI and scenario

*Note: different scales for each BMI strata*

# REFERENCES

[1 Clark RA, Weerasuriya CK, Portnoy A, *et al.* New tuberculosis vaccines in India: Modelling the potential health and economic impacts of adolescent/adult vaccination with M72/AS01E and BCG-revaccination. *BMC Med* 2023; **21**. DOI:https://doi.org/10.1186/s12916-023-02992-7.](https://www.zotero.org/google-docs/?SIztxb)

[2 Rajagopalan S. Tuberculosis and aging: a global health problem. *Clin Infect Dis Off Publ Infect Dis Soc Am* 2001; **33**: 1034–9.](https://www.zotero.org/google-docs/?SIztxb)

3 [Prem K, Zandvoort K van, Klepac P, *et al.* Projecting contact matrices in 177 geographical regions: An update and comparison with empirical data for the COVID-19 era. *PLOS Comput Biol* 2021; **17**: e1009098.](https://www.zotero.org/google-docs/?SIztxb)

[4 Abu-Raddad L, Sabatelli L, Achterberg JT, *et al.* Epidemiological benefits of more-effective tuberculosis vaccines, drugs, and diagnostics. *Proc Natl Acad Sci U S A* 2009; **106**: 13980–5.](https://www.zotero.org/google-docs/?SIztxb)

[5 Dye C, Williams BG. Eliminating human tuberculosis in the twenty-first century. *J R Soc Interface* 2008; **5**: 653–62.](https://www.zotero.org/google-docs/?SIztxb)

[6 Sutherland I, Svandová E, Radhakrishna S. The development of clinical tuberculosis following infection with tubercle bacilli. 1. A theoretical model for the development of clinical tuberculosis following infection, linking from data on the risk of tuberculous infection and the incidence of clinical tuberculosis in the Netherlands. *Tubercle* 1982; **63**: 255–68.](https://www.zotero.org/google-docs/?SIztxb)

[7 Vynnycky E, Fine PE. The natural history of tuberculosis: the implications of age-dependent risks of disease and the role of reinfection. *Epidemiol Infect* 1997; **119**: 183–201.](https://www.zotero.org/google-docs/?SIztxb)

[8 Gabriela M. Gomes M, Rodrigues P, Hilker FM, *et al.* Implications of partial immunity on the prospects for tuberculosis control by post-exposure interventions. *J Theor Biol* 2007; **248**: 608–17.](https://www.zotero.org/google-docs/?SIztxb)

[9 United Nations, Department of Economic and Social Affairs, Population Division. World Population Projections [2019 Revision]. 2019. https://population.un.org/wpp/Download/Standard/Population/ (accessed Nov 2, 2022).](https://www.zotero.org/google-docs/?SIztxb)

[10 Tiemersma EW, Werf MJ van der, Borgdorff MW, Williams BG, Nagelkerke NJD. Natural History of Tuberculosis: Duration and Fatality of Untreated Pulmonary Tuberculosis in HIV Negative Patients: A Systematic Review. *PLOS ONE* 2011; **6**: e17601.](https://www.zotero.org/google-docs/?SIztxb)

[11 Quaife M, Houben RMGJ, Allwood B, *et al.* Post-tuberculosis mortality and morbidity: valuing the hidden epidemic. *Lancet Respir Med* 2020; **8**: 332–3.](https://www.zotero.org/google-docs/?SIztxb)

[12 World Health Organization. WHO TB burden estimates. CSV Files Download. 2022. https://www.who.int/tb/country/data/download/en/ (accessed Nov 2, 2022).](https://www.zotero.org/google-docs/?SIztxb)

[13 World Health Organization. Case Notifications. CSV Files Download. 2022. https://www.who.int/tb/country/data/download/en/ (accessed Nov 2, 2022).](https://www.zotero.org/google-docs/?SIztxb)

[14 Emery J. Subclinical TB disease: lessons and questions from field to lab. 2020.](https://www.zotero.org/google-docs/?SIztxb)

[15 Emery JC, Richards AS, Dale KD, *et al.* Self-clearance of *Mycobacterium tuberculosis* infection: implications for lifetime risk and population at-risk of tuberculosis disease. *Proc R Soc B Biol Sci* 2021; **288**: 20201635.](https://www.zotero.org/google-docs/?SIztxb)

[16 Marx FM, Dunbar R, Enarson DA, *et al.* The Temporal Dynamics of Relapse and Reinfection Tuberculosis After Successful Treatment: A Retrospective Cohort Study. *Clin Infect Dis* 2014; **58**: 1676–83.](https://www.zotero.org/google-docs/?SIztxb)

[17 Gomes MGM, Franco AO, Gomes MC, Medley GF. The reinfection threshold promotes variability in tuberculosis epidemiology and vaccine efficacy. *Proc R Soc B Biol Sci* 2004; **271**: 617–23.](https://www.zotero.org/google-docs/?SIztxb)

[18 Dangisso MH, Woldesemayat EM, Datiko DG, Lindtjørn B. Long-term outcome of smear-positive tuberculosis patients after initiation and completion of treatment: A ten-year retrospective cohort study. *PloS One* 2018; **13**: e0193396.](https://www.zotero.org/google-docs/?SIztxb)

[19 Global Health Observatory. https://www.who.int/data/gho (accessed Dec 19, 2023).](https://www.zotero.org/google-docs/?SIztxb)

[20 International Institute for Population Sciences (IIPS) and ICF. National Family Health Survey (NFHS-3), 2005-06. Mumbai, 2007.](https://www.zotero.org/google-docs/?SIztxb)

[21 International Institute for Population Sciences (IIPS) and ICF. National Family Health Survey (NFHS-4), 2015-16. Mumbai, 2017.](https://www.zotero.org/google-docs/?SIztxb)

[22 International Institute for Population Sciences (IIPS) and ICF. National Family Health Survey (NFHS-5), 2019-21. Mumbai, 2022.](https://www.zotero.org/google-docs/?SIztxb)

[23 Lumley T. survey: Analysis of Complex Survey Samples. 2023; published online May 3. https://cran.r-project.org/web/packages/survey/index.html (accessed Feb 5, 2024).](https://www.zotero.org/google-docs/?SIztxb)

24 Lönnroth K, Williams BG, Cegielski P, Dye C. A consistent log-linear relationship between tuberculosis incidence and body mass index. *Int J Epidemiol* 2010; 39: 149–55.

25 Cegielski JP, Arab L, Cornoni-Huntley J. Nutritional Risk Factors for Tuberculosis Among Adults in the United States, 1971–1992. *Am J Epidemiol* 2012; **176**: 409–22.

[26 Sinha P, Ponnuraja C, Gupte N, *et al.* Impact of Undernutrition on Tuberculosis Treatment Outcomes in India: A Multicenter, Prospective, Cohort Analysis. *Clin Infect Dis* 2023; **76**: 1483–91.](https://www.zotero.org/google-docs/?SIztxb)

[27 Andrianakis I, Vernon I, McCreesh N, *et al.* History matching of a complex epidemiological model of human immunodeficiency virus transmission by using variance emulation. *J R Stat Soc Ser C Appl Stat* 2017; **66**: 717–40.](https://www.zotero.org/google-docs/?SIztxb)

[28 Andrianakis I, Vernon IR, McCreesh N, *et al.* Bayesian History Matching of Complex Infectious Disease Models Using Emulation: A Tutorial and a Case Study on HIV in Uganda. *PLOS Comput Biol* 2015; **11**: e1003968.](https://www.zotero.org/google-docs/?SIztxb)

[29 Goldstein M. Bayes Linear Analysis for Complex Physical Systems Modeled by Computer Simulators. In: Dienstfrey AM, Boisvert RF, eds. Uncertainty Quantification in Scientific Computing. Berlin, Heidelberg: Springer Berlin Heidelberg, 2012: 78–94.](https://www.zotero.org/google-docs/?SIztxb)

[30 Williamson D, Goldstein M, Allison L, *et al.* History matching for exploring and reducing climate model parameter space using observations and a large perturbed physics ensemble. *Clim Dyn* 2013; **41**: 1703–29.](https://www.zotero.org/google-docs/?SIztxb)

[31 Iskauskas A. hmer: History Matching and Emulation Package. 2022. https://CRAN.R-project.org/package=hmer (accessed Nov 2, 2022).](https://www.zotero.org/google-docs/?SIztxb)

[32 Iskauskas A, Vernon I, Goldstein M, *et al.* Emulation and History Matching using the hmer Package. 2022; published online Sept 12. http://arxiv.org/abs/2209.05265 (accessed Dec 20, 2022).](https://www.zotero.org/google-docs/?SIztxb)

[33 Jabot F, Faure T, Dumoulin N. EasyABC: performing efficient approximate Bayesian computation sampling schemes using R. *Methods Ecol Evol* 2013; **4**: 684–7.](https://www.zotero.org/google-docs/?SIztxb)

[34 Roberts GO, Rosenthal JS. Examples of Adaptive MCMC. *J Comput Graph Stat* 2009; **18**: 349–67.](https://www.zotero.org/google-docs/?SIztxb)

35 World Health Organization. WHO TB incidence estimates disaggregated by age group, sex and risk factor. CSV files to download. Published 2022. Accessed November 2, 2022. https://www.who.int/tb/country/data/download/en/

36 Pandey S, Chadha VK, Laxminarayan R, Arinaminpathy N. Estimating tuberculosis incidence from primary survey data: a mathematical modeling approach. Int J Tuberc Lung Dis. 2017;21(4):366-374. doi:10.5588/ijtld.16.0182

[37 Indian Council of Medical Research (ICMR). National TB prevalence survey India 2019-2021. 2022.](https://www.zotero.org/google-docs/?SIztxb)

[38 Frascella B, Richards AS, Sossen B, *et al.* Subclinical tuberculosis disease - a review and analysis of prevalence surveys to inform definitions, burden, associations and screening methodology. *Clin Infect Dis* 2021; **73**: e830–41.](https://www.zotero.org/google-docs/?SIztxb)

[39 Smith B, Wårlind D, Arneth A, *et al.* Implications of incorporating N cycling and N limitations on primary production in an individual-based dynamic vegetation model. *Biogeosciences* 2014; **11**: 2027–54.](https://www.zotero.org/google-docs/?SIztxb)

[40 Alexander P, Rabin S, Anthoni P, *et al.* Adaptation of global land use and management intensity to changes in climate and atmospheric carbon dioxide. *Glob Change Biol* 2018; **24**: 2791–809.](https://www.zotero.org/google-docs/?SIztxb)

[41 Gouel C, Guimbard H. Nutrition Transition and the Structure of Global Food Demand. *Am J Agric Econ* 2019; **101**: 383–403.](https://www.zotero.org/google-docs/?SIztxb)

[42 Taylor KE, Stouffer RJ, Meehl GA. An Overview of CMIP5 and the Experiment Design. *Bull Am Meteorol Soc* 2012; **93**: 485–98.](https://www.zotero.org/google-docs/?SIztxb)

[43 Dufresne J-L, Foujols M-A, Denvil S, *et al.* Climate change projections using the IPSL-CM5 Earth System Model: from CMIP3 to CMIP5. *Clim Dyn* 2013; **40**: 2123–65.](https://www.zotero.org/google-docs/?SIztxb)

[44 Springmann M, Wiebe K, Mason-D’Croz D, Sulser TB, Rayner M, Scarborough P. Health and nutritional aspects of sustainable diet strategies and their association with environmental impacts: a global modelling analysis with country-level detail. *Lancet Planet Health* 2018; **2**: e451–61.](https://www.zotero.org/google-docs/?SIztxb)

[45 Alexander P, Arneth A, Henry R, Maire J, Rabin S, Rounsevell MDA. High energy and fertilizer prices are more damaging than food export curtailment from Ukraine and Russia for food prices, health and the environment. *Nat Food* 2023; **4**: 84–95.](https://www.zotero.org/google-docs/?SIztxb)

[46 O’Neill BC, Kriegler E, Riahi K, *et al.* A new scenario framework for climate change research: the concept of shared socioeconomic pathways. *Clim Change* 2014; **122**: 387–400.](https://www.zotero.org/google-docs/?SIztxb)

[47 van Vuuren DP, Edmonds J, Kainuma M, *et al.* The representative concentration pathways: an overview. *Clim Change* 2011; **109**: 5.](https://www.zotero.org/google-docs/?SIztxb)

[48 World Health Organization. WHO TB burden estimates. CSV Files Download. 2023. https://www.who.int/tb/country/data/download/en/ (accessed Dec 19, 2023).](https://www.zotero.org/google-docs/?SIztxb)
